# Diverse interventions that extend mouse lifespan suppress shared age-associated epigenetic changes at critical gene regulatory regions

**DOI:** 10.1186/s13059-017-1185-3

**Published:** 2017-03-28

**Authors:** John J. Cole, Neil A. Robertson, Mohammed Iqbal Rather, John P. Thomson, Tony McBryan, Duncan Sproul, Tina Wang, Claire Brock, William Clark, Trey Ideker, Richard R. Meehan, Richard A. Miller, Holly M. Brown-Borg, Peter D. Adams

**Affiliations:** 1 Beatson Institute for Cancer Research and University of Glasgow, Garscube Estate, G61 1BD UK; 2 Department of Pathology and Glenn Center for the Biology of Aging, University of Michigan, Ann Arbor, MI 48109 USA; 3 MRC Human Genetics Unit, Institute of Genetics and Molecular Medicine, Western General Hospital, Crewe Road, Edinburgh, UK; 4 Edinburgh Cancer Research Centre, Institute of Genetics and Molecular Medicine, Western General Hospital, Crewe Road, Edinburgh, UK; 5 Department of Biomedical Sciences, University of North Dakota School of Medicine and Health Sciences, Grand Forks, ND 58203 USA; 6 Department of Medicine, University of California San Diego, La Jolla, CA 92093 USA; 7 Sanford Burnham Prebys Medical Discovery Institute, 10901 North Torrey Pines Road, La Jolla, CA 92037 USA

## Abstract

**Background:**

Age-associated epigenetic changes are implicated in aging. Notably, age-associated DNA methylation changes comprise a so-called aging “clock”, a robust biomarker of aging. However, while genetic, dietary and drug interventions can extend lifespan, their impact on the epigenome is uncharacterised. To fill this knowledge gap, we defined age-associated DNA methylation changes at the whole-genome, single-nucleotide level in mouse liver and tested the impact of longevity-promoting interventions, specifically the Ames dwarf *Prop1* ^*df/df*^ mutation, calorie restriction and rapamycin.

**Results:**

In wild-type mice fed an unsupplemented ad libitum diet, age-associated hypomethylation was enriched at super-enhancers in highly expressed genes critical for liver function. Genes harbouring hypomethylated enhancers were enriched for genes that change expression with age. Hypermethylation was enriched at CpG islands marked with bivalent activating and repressing histone modifications and resembled hypermethylation in liver cancer. Age-associated methylation changes are suppressed in Ames dwarf and calorie restricted mice and more selectively and less specifically in rapamycin treated mice.

**Conclusions:**

Age-associated hypo- and hypermethylation events occur at distinct regulatory features of the genome. Distinct longevity-promoting interventions, specifically genetic, dietary and drug interventions, suppress some age-associated methylation changes, consistent with the idea that these interventions exert their beneficial effects, in part, by modulation of the epigenome. This study is a foundation to understand the epigenetic contribution to healthy aging and longevity and the molecular basis of the DNA methylation clock.

**Electronic supplementary material:**

The online version of this article (doi:10.1186/s13059-017-1185-3) contains supplementary material, which is available to authorized users.

## Background

Genetic, dietary and drug interventions can enhance longevity and suppress age-associated disease, such as cancer. Prominent genetic interventions that robustly extend longevity and healthspan in mammals include those that decrease growth hormone (GH) and insulin-like growth factor (IGF) signalling; for example, Ames dwarf mice live more than 50% longer than their wild-type siblings [1]. These diminutive mice result from a point mutation in a gene (*Prop1* ^*df/df*^) that drives development of the pituitary gland, so that mutant mice are deficient in specific hormones. The GH deficiency, in particular, has been shown to underlie their enhanced health span and extended lifespan. Ames mice are highly insulin-sensitive, resistant to some stresses and the incidence of cancer is delayed [2–4]. Dietary and drug interventions that extend lifespan include calorie restriction (CR) and the mTOR inhibitor rapamycin [5]. Like the Ames dwarf mutation, CR and rapamycin also suppress and/or delay the incidence of cancer [5–7]. A detailed understanding of how these interventions exert their beneficial effects is essential to develop strategies to promote healthy aging in humans [8]. Currently, these interventions are thought to exert their effects by related and interconnected effects on some or all of the following: genome stability, the epigenome, telomere attrition and/or function, protein quality control, mitochondrial function, nutrient sensing, cellular senescence, stem cell exhaustion, cellular stress responses and altered intercellular communication [9]. Of note, the effects of longevity promoting interventions on the epigenome, a key determinant of cell phenotype, are poorly understood.

Aging is associated with changes to the epigenome [10, 11]. These changes include age-associated accumulation of histone variants, for example histone H3.3 in neurons and macroH2A in lung, liver and muscle, as well as other chromatin-associated proteins and changes to histone and DNA modifications [12–14]. Aging also affects specific gene regulatory elements, such as enhancers, promoters and CpG islands [15–23]. Underscoring the importance of such age-associated epigenetic changes, recent human studies have identified collections of specific CpGs whose age-associated change in methylation status in multiple tissues correlates strongly with chronological age. An advanced methylation age compared to actual chronological age is thought to reflect accelerated biological age and is linked to increased mortality [24–28].

Age-associated epigenetic changes are not just biomarkers or passengers in the aging process, but can be causative in control of lifespan [29–31]. For example, in yeast, accumulation of H4K16ac at subtelomeric regions promotes replicative aging, while inactivation of the chromatin remodeler Iswi2p or the H3K36me2/3 demethylase Rph1p extends lifespan [29, 32, 33]. Decreased H3K4 methyltransferase activity can extend worm lifespan in a germline dependent manner [30]. In mice, muscle stem cells from old mice exhibit elevated repressive H3K27me3 at repressed histone genes [34], perhaps responsible for decreased proliferative potential of aged stem cells compared to young stem cells [35]. Mouse haematopoietic stem cells (HSCs) also exhibit changes in DNA methylation with age, including a small net hypermethylation both globally and at CpG islands [36, 37]. Some of these changes in aged cells are thought to promote expression of self-renewal genes and impair expression of differentiation genes, including lymphoid genes. This can contribute to the characteristic phenotypes of aged HSCs, such as increased number, decreased function and a predisposition to myeloid differentiation [36].

Epigenetic changes linked to aging also impact specific diseases of aging, including cancer. While some age-associated epigenetic changes, such as increased abundance of histone modification H4K20me3 [10] and decreased H3K27me3 [38, 39], may activate tumour suppressor mechanisms and prevent cancer, others may be tumour promoting. Like cancer, aged tissue has been reported to exhibit global DNA hypomethylation and more focal hypermethylation at CpG islands [10]. Most notably, so-called bivalent gene promoters, marked with both activating H3K4me3 and repressing H3K27me3 (hence “bivalent”) in embryonal stem (ES) cells, acquire DNA methylation in aged tissues and are also methylated and stably silenced in cancer [15–19]. In ES cells, these bivalent-marked genes are thought to be poised for activation due to loss of the repressive H3K27me3 mark during stem and progenitor cell differentiation and development. By virtue of their pro-differentiation functions these genes tend to have tumour suppressor-like properties, meaning that their methylation and stable silencing may promote proliferation, self-renewal and malignancy. In the haematopoietic system, some CpG islands progressively increase methylation from young to old to neoplasia, namely myelodysplastic syndrome (MDS) and ultimately acute myeloid leukemia [40]. *Sf3b1*, the mouse ortholog of a gene frequently mutated in human MDS, is methylated and underexpressed in aged mouse HSCs [36]. Hence, age-associated methylation changes might predispose to transformation of aged cells by promoting silencing of tumour suppressor genes.

Given this strong and accumulating evidence that epigenetic events are important determinants of lifespan and predisposition to disease, we set out to ask whether genetic, dietary and drug interventions that promote healthy aging and longevity suppress age-associated DNA methylation changes.

## Methods

Ames dwarf mice were derived from a closed colony with a heterogeneous background (over 25 years) at the University of North Dakota [41]. Dwarf mice (and corresponding wild type (WT)) were generated by mating either homozygous (df/df) or heterozygous (df/+) dwarf males with heterozygous females (df/+). Non-genotypic intervention studies (rapamycin and CR) utilised genetically heterogeneous WT UM-HET3 mice bred at the University of Michigan. One cohort was given encapsulated rapamycin (42 parts per million (ppm)) from 4 months of age, and another group a CR diet initiated at 4 months of age (these mice received 60% of the intake of their age-matched controls after a two week run-in period at 80%). All cohorts contained four replicates (four mice). Liver tissue was collected at 2 and 22 months of age and DNA isolated using a DNeasy Blood and Tissue Kit (Qiagen). Hepatocellular carcinoma (HCC) is a relatively common disease of aging in mice. Hence, to avoid distortion of our data by neoplastic tissue, aged mice were sacrificed at 22 months (before HCC is typically apparent) and livers with overt signs of neoplasia were excluded from analysis. Where whole-genome bisulphite sequencing (WGBS-seq) was performed (by BGI, Shenzen), samples underwent a standard protocol of sonication, DNA-end repair and ligation of methylated adapter sequences prior to bisulphite conversion using a ZYMO EZ DNA Methylation-Gold Kit (Zymo Research) and then 90-bp, paired-end sequencing on the Illumina Hi-Seq 4000 platform. Sequenced reads were aligned to the reference genome (mm9) and methylation status of CpGs determined using Bismark and Bowtie2 [42, 43]. The bioinformatics process involved read quality assessment via FastQC, read trimming using the package Trim-Galore with alignment, read de-duplication and methylation context extraction via the Bismark suite [42]. CpG dyads were collapsed by combining the methylated and unmethylated scores at each dyad locus. The mouse genome contains approximately 42 million CpG loci (mm9)—or 21.3 million CpG dyads. We achieved sufficient coverage to represent between 94 and 96% of the dyads in the young and old, WT and Ames dwarf dataset, with a mean coverage of 6.96 methylation calls per site (four biological replicates per cohort (approximately 15-fold coverage per genome), to yield approximately 1500 Gbp of data (Additional file 1: Table S1)). Despite sequencing CR and rapamycin intervention data with reduced coverage, we still observe 86–89% of all mappable CpG dyads with a mean coverage of 4.37 reads per loci. To identify differentially methylated CpG sites, a two-tailed Fisher exact test (FET) was used with *p* value correction using a Benjimini–Hochberg (BH)-false discovery rate (FDR) function at a rate of 5% where coverage surpassed a threshold of ten overlapping reads. To identify differentially methylated regions (DMRs), we used a sliding window-based approach operating at a range of 500 bp. At each window, a two-tailed FET was performed to determine DMR significance alongside a chi-squared test of heterogeneity across the four mouse replicates within each cohort. Both chi-squared and FET tests were multi-sample corrected using BH-FDR with DMRs selected on the basis of significant (*p* < 0.05) BH-FDR-corrected FET score and non-significant intra-cohort heterogeneity via the FDR-corrected chi-squared test. We then divided DMRs into hyper- and hypomethylated based on positive or negative changes in their respective methylation relative to their control. To determine the significance of the overlaps between regions or features, we used a permutation-based approach to assess significant enrichment over equally sized, randomly generated regions and calculated fold enrichments based on how these regions overlap compared to an expected (random) model level of intersection. We validated findings using MeDIP-seq, an enrichment based assay that enriches methylated DNA fragments via immunoprecipitation with anti-methyl-cytosine antibodies [44]. Additional details, including RNA- and ChIP-seq analysis information, are available in Additional file 2: Supplementary methods.

## Results

### The epigenomes of wild-type and Ames dwarf mice diverge with age

**Fig. 1:**
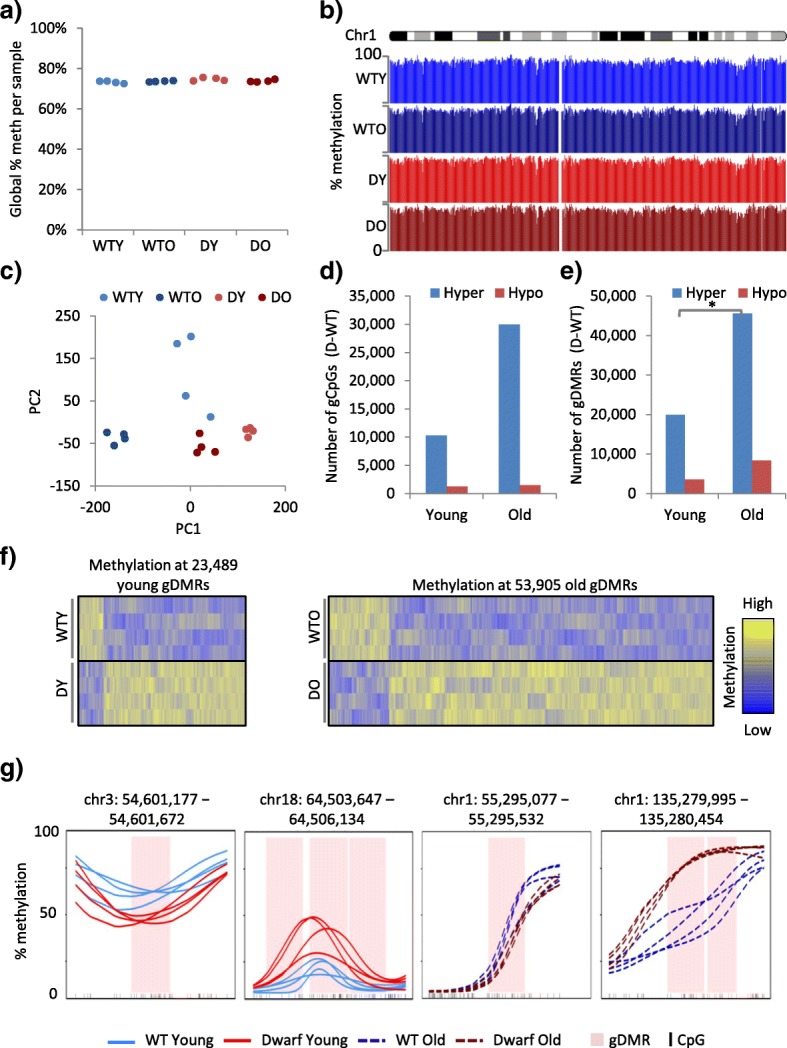
The epigenomes of wild-type and Ames dwarf mice diverge with age. **a** Global percentage methylation per liver sample, for 2-month-old wild-type (*WTY*), 22-month-old wild-type (*WTO*), 2-month-old Ames dwarf (*DY*) and 22-month-old Ames dwarf (*DO*) mice. All *p* > 0.05 (two tailed *t*-test on arcsine transformed proportions). **b** UCSC genome browser trace of percentage methylation over chromosome 1, showing pooled WTY (*light blue*), WTO (*dark blue*), DY (*light red*), DO (*dark red*) replicate tracks. **c** Principal component analysis of CpG percentage methylation, for WTY (*light blue*), WTO (*dark blue*), DY (*light red*) and DO (*dark red*) liver samples. Principal component (PC)1 proportion of variance = 12.38%, and PC2 proportion of variance = 7.64%. **d** The number of significantly differentially methylated CpGs (5% FDR, Fisher’s exact test) between four pooled WT and four pooled Ames dwarf replicates (*gCpGs*) in liver of 2-month-old (young) and 22-month-old (old) mice. Hyper- and hypomethylated gCpGs are higher and lower in Ames dwarf mice, respectively. **e** As **d** but showing significantly differentially methylated regions (*gDMRs*; 5% FDR, Fisher’s exact test, 500-bp windows). Regions of heterogeneity (chi-squared test <0.05) across the four replicates in each cohort were removed. DY-WTY versus DO-WTO hypermethylation, *p* < 0.05 (marked with an asterisk). See also Additional file 3: Table S1e. **f** The percentage methylation across all 2-month-old (*left*) and 22-month-old (*right*) gDMRs. Replicate samples (four mouse livers) are in rows and the gDMRs in columns. The intensity of the heatmap represents column scaled percentage methylation (Z-score), with values ranging from lower to higher methylation shown as *blue* to *yellow*. **g** Kernel smoothed line plots of selected gDMRs, ±5 kb. WTY, DY, WTO and DO replicates are represented by *solid blue*, *solid red*, *dashed blue* and *dashed red lines*, respectively. DMRs are highlighted in *pink* and CpGs in *black*

To investigate the relationship between age-associated epigenetic changes and healthy aging and longevity, we first set out to compare the DNA methylome of liver from young and old male WT and long-lived Ames dwarf mice. We selected liver for this study because our previous studies showed differences in liver in expression of DNA methyltransferases (DNMTs) between WT and Ames mice and young and old mice [45]. Also, a single cell type, the hepatocyte, comprises ~80% of liver mass, and the epigenome of mouse liver has been extensively characterised, thereby aiding downstream analysis of the methylome in the context of the wider epigenetic landscape. We performed WGBS-seq on the livers of young adult (2 months of age) and old (22 months of age) Ames dwarf and WT mice. We found that global levels of methylation across all CpGs in the genome were highly similar between all age groups, mouse genotypes and replicates (Fig. 1a). This was also apparent from viewing whole-chromosome methylation profiles on the UCSC genome browser (Fig. 1b). However principal component analysis (PCA) of the data (percentage methylation per CpG) suggested that local differences in methylation were present, as cohorts separated well on the first and/or second principal components (12.38 and 7.64% of the respective cumulative variance) (Fig. 1c).

Previous studies showed that epigenomes become more divergent with age [46]. Therefore, we set out to assess differences between WT and Ames dwarf mice at 2 months and 22 months’ of age. We determined those CpGs differentially methylated between genotypes (i.e. between WT and Ames dwarf mice (gCpGs)). This was performed separately in both the young and old mice, using a Fisher’s exact test method at 5% FDR (“Methods”). In dwarf mice, compared to WT mice, approximately ten times more CpGs were hypermethylated than were hypomethylated (Fig. 1d). Strikingly, we observed a larger number of significantly hypermethylated gCpGs (Fig. 1d) in the older mice compared to the young, suggesting that the WT and Ames mice exhibit more epitype differences with age. These differences were also visualised by identifying all gDMRs for both the young and the old mice using a dynamic 500-bp sliding window and Fisher’s exact test method at 5% FDR and also removing any DMRs that were not consistent across all four replicates (“Methods”; Fig. 1e; Additional file 3: Table S1e). We observed a significant increase in hypermethylated gDMRs, while the increase in hypomethylated gDMRs was not significant (Fig. 1e; Additional file 3: Table S1e). The consistency of the gDMRs across all four replicates within each cohort was confirmed in both gDMR heatmaps (Fig. 1f) and kernel smoothed methylation plots of representative gDMRs (Fig. 1g). Together, these data establish that between the Ames and WT mice there are more than 20,000 DMRs, and the number of these epigenotype differences increases markedly with age.

### The Ames dwarf epigenome appears more stable and buffered against age-associated hypomethylation

**Fig. 2:**
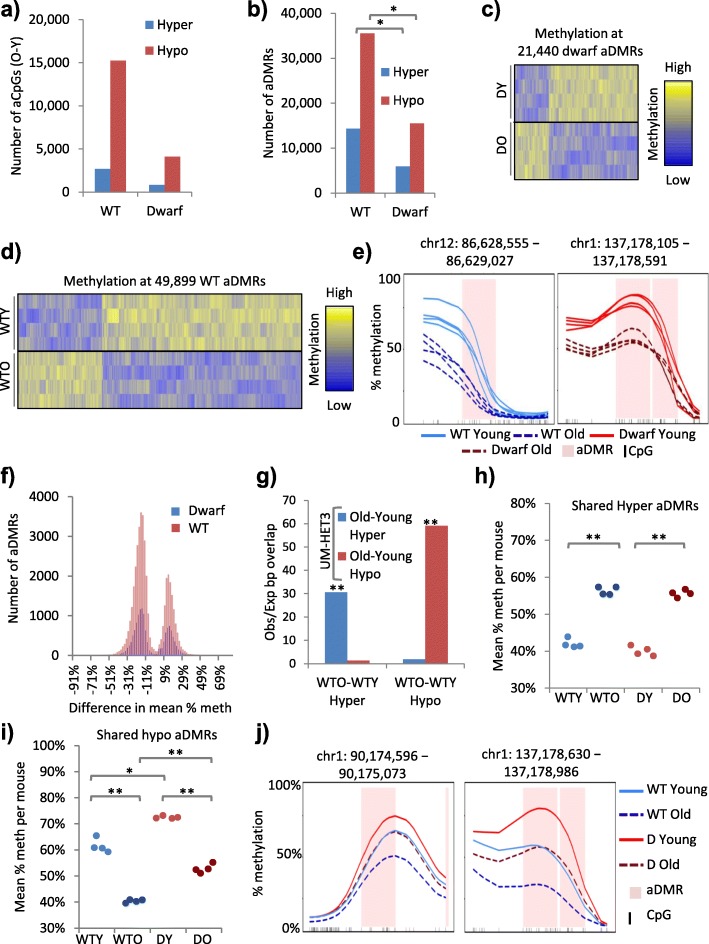
The Ames dwarf epigenome appears more stable and buffered against age-associated hypomethylation. **a** The number of significantly differentially methylated CpGs (5% FDR, Fisher’s exact test) between 2 and 22-month-old WT (*WT*) and between 2- and 22-month-old Ames dwarf (*Dwarf*) mice. Hyper- and hypomethylated aCpGs are higher and lower in 22-month-old mice, respectively. **b** The number of significantly differentially methylated regions (aDMRs; 5% FDR, Fisher’s exact test, 500-bp windows) between 2- and 22-month-old mice for WT and Ames dwarf mice. Regions of heterogeneity (chi-squared test <0.05) across the four replicates in each cohort were removed. Significance (empirical *p* value) at *p* < 0.05 is indicated with an asterisk. See also Additional file 3. **c** The percentage methylation across all 2- (*DY*) versus 22-month-old (*DO*) Ames dwarf differentially methylated regions (*aDMRs*). Replicate samples (four mouse livers) are in rows and the aDMRs in columns. The intensity of the heatmap represents column scaled percentage methylation (Z-score), with values ranging from lower to higher methylation shown as *blue* to *yellow*. **d** The percentage methylation across all 2- (*WTY*) versus 22-month-old (*WTO*) WT differentially methylated regions (*aDMRs*). Replicate samples (four mouse livers) are in rows and the aDMRs in columns. Columns are scaled using Z-scores. The intensity of the heatmap represents Z-score, with values ranging from negative to positive shown as *blue* to *yellow*. **e** Kernel smoothed line plots of selected aDMRs, ±5 kb. WTY, 2-month-old dwarf (DY), WTO and 22-month-old dwarf (DO) replicates are represented by *solid blue*, *solid red*, *dashed blue* and *dashed red lines*, respectively. DMRs are highlighted in pink and CpGs in black. **f** The difference in mean percentage methylation per DMR (across all samples) between 22- and 2-month-old mice versus number of aDMRs. WT aDMRs are shown in *red* and dwarf aDMRs in *blue*. **g** Ratio of observed/expected (random) overlap between WT (from **a**–**d**) and UM-HET3 aDMRs. Hyper- and hypomethylated aDMRs are higher and lower in old mice, respectively. Significance (empirical *p* value) at *p* < 0.001 is indicated with double asterisks. **h** Mean percentage methylation per replicate across all hypermethylated aDMRs common to both WT and Ames dwarf mice (shared). WTY, WTO, DY and DO mice are shown in *light blue*, *dark blue*, *light red* and *dark red*, respectively. WTY versus WTO and DY versus DO at *p* < 0.001 are indicated with double asterisks (two tailed *t*-test on arcsine transformed proportions). **i** As **h** but showing shared hypomethylated aDMRs. WTY versus WTO, DY versus DO, WTO versus DO all *p* < 0.001 indicated with double asterisks and WTY versus DY *p* < 0.05 indicated with a single asterisk (two tailed *t*-test on arcsine transformed proportions). **j** Kernel smoothed line plots of selected aDMRs common to both WT and dwarf mice, ±5 kb. Pooled replicates for WTY, DY, WTO and DO are represented by *solid blue*, *solid red*, *dashed blue* and *dashed red lines*, respectively. DMRs are highlighted in *pink* and CpGs in *black*

To further investigate these age-associated epigenotype differences between the WT and dwarf mice, we characterised age-associated differentially methylated CpGs (i.e. CpGs whose methylation status changes with age in either WT or Ames dwarf mice (aCpGs)) in both the WT and dwarf mice using a Fisher’s exact test method at 5% FDR (“Methods”). Strikingly, we observed roughly three times more significant aCpGs in the WT than the dwarf mice (Fig. 2a), suggesting that the methylome of dwarf mice is more stable through chronological aging. Similarly, we detected less than half the number of aDMRs in dwarf mice compared to WT mice (Fig. 2b; Additional file 3: Table S2b). Changes in the aDMRs were consistent across all four replicates within each cohort, as confirmed by aDMR heatmaps (Fig. 2c, d) and representative kernel smoothed methylation plots (Fig. 2e). Although we observed significantly more aDMRs in the WT than the dwarf mice, the magnitude of the methylation change per DMR was comparable in the WT and dwarf mice (Fig. 2f). To confirm these aDMR loci in other mouse cohorts and strains, we performed WGBS-seq of whole liver from 2-month-old and 22-month-old female UM-HET3 mice from the NIA Intervention Testing Program (ITP) cohorts (four mouse replicates per age group, approximately 5× coverage per replicate) (Additional file 4: Table S2). Global methylation was comparable to Ames dwarf and corresponding WT and, again, between young and old (compare Fig. 1a and Additional file 5: Figure S1a). However, PCA again separated the young and old UM-HET3 mice, indicative of their differential methylation (Additional file 5: Figure S1b). Hence, aDMRs were identified between young and old UM-HET3 mice (“Methods”). The extent of overlap of the two sets of WT hypomethylated aDMRs (i.e. the WT used for comparison to Ames dwarf throughout and WT UM-HET3 (see “Methods”)) was much greater than expected from random overlap and highly significant. The same was the case for the two sets of hypermethylated aDMRs. However, there was minimal overlap between hypermethylated and hypomethylated aDMRs (Fig. 2g). In sum, aging of the liver generates thousands of discrete aDMRs. Significantly, the epigenome of WT mice exhibits many more such regions than that of Ames dwarf mice, suggesting that the Ames dwarf epigenome is more stable with chronological age than the WT epigenome.

While many aDMRs were restricted to WT mice, other aDMRs were restricted to Ames mice or were shared by both genotypes (Additional file 5: Figure S1c). Plots of mean percentage methylation per mouse liver sample at each subset of DMR (i.e. WT hypermethylated aDMRs (Additional file 5: Figure S1d), dwarf hypermethylated aDMRs (Additional file 5: Figure S1e) and hypermethylated aDMRs shared between WT and dwarf (Fig. 2h) confirmed the DMR subsets and consistency between mouse replicates (Fig. 2h, i; Additional file 5: Figure S1d–g). For example, at shared hypermethylated aDMRs, methylation increased comparably with age in both WT and dwarf mice (Fig. 2h). The methylation changes at hypomethylated aDMRs were particularly interesting. At the hypomethylated aDMRs restricted to WT mice, the young and old Ames mice both showed methylation comparable to the young WT mice (Additional file 5: Figure S1f), while at the hypomethylated aDMRs restricted to the Ames mice, the young and old WT mice both showed methylation comparable to the older Ames mice (Additional file 5: Figure S1g). At shared hypomethylated aDMRs, while the magnitude of the change was comparable between WT and Ames, the dwarf mice showed consistently higher methylation than the WT in both age groups (Fig. 2i). This phenomenon was also apparent in the population of individual aDMRs, which showed higher methylation in dwarf in both young and old mice (i.e. below right of the 45° diagonal in both plots) and an age-associated loss of methylation in both WT and dwarf (i.e. closer to zero on both x and y axes in old mice) (Additional file 5: Figure S1h). At representative shared hypomethylated aDMRs, methylation declined with age in both WT and dwarf mice but began at higher levels in the young dwarf (Fig. 2j). In sum, in dwarf mice, hypomethylated aDMRs were biased towards a higher methylation level. This is most notable at hypomethylated aDMRs shared between WT and Ames dwarf mice, where the latter exhibited a higher initial level of methylation in young animals, thus potentially buffering them against the effects of age-associated hypomethylation.

### Hypomethylated aDMRs are enriched at intragenic enhancers in highly expressed liver-specific genes

**Fig. 3:**
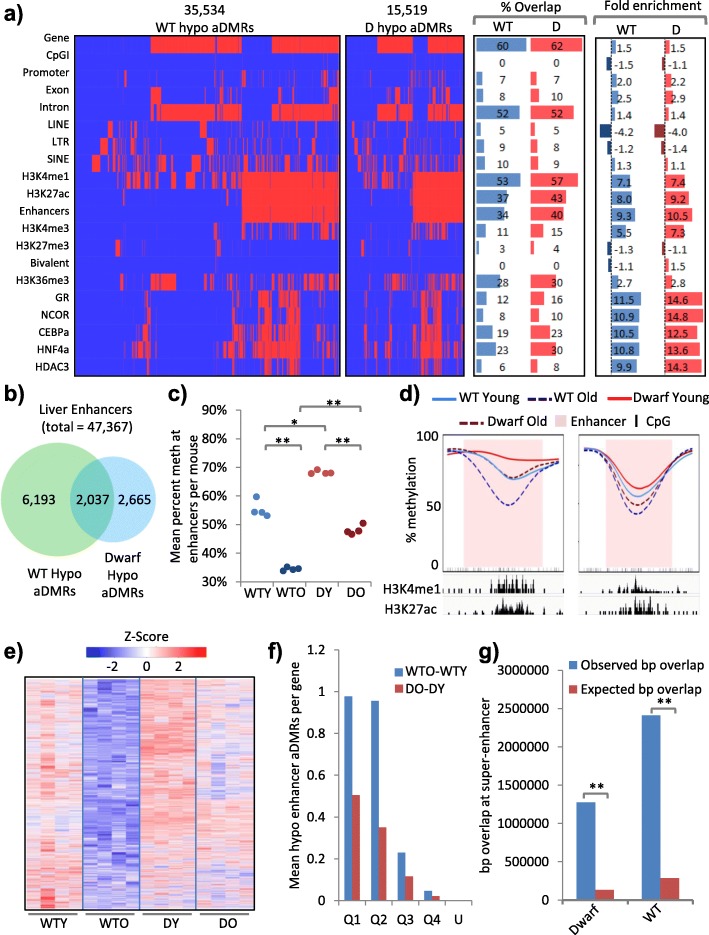
Hypomethylated aDMRs are enriched at intragenic enhancers in highly expressed liver-specific genes. **a** Clustered feature interaction maps of spatial overlap between hypomethylated aDMRs (*columns*) and a selection of genomic, histone and transcription factor features (*rows*), showing WT (*WT*; *left*) and dwarf (*D*; *centre left*) aDMRs. *Red* indicates an overlap between an aDMR and a feature and *blue* no overlap. Interaction map x-axes are scaled by number of aDMRs. The percentage overlap (*centre right*) and fold enrichment observed/expected (random) overlap (*right*; units of fold) for each feature are given. **b** The overlap between enhancers that contain hypomethylated aDMRs in WT and Ames dwarf mice. Enrichment of overlap observed/expected 153-fold, *p* < 0.001. **c** Mean percentage methylation per replicate across enhancers that contain hypomethylated aDMRs in both WT and dwarf mice. For 2-month-old WT (*WTY*; *light blue*), 22-month-old WT (*WTO*; *dark blue*), 2-month-old dwarf (*DY*; *light red*) and 22-month-old dwarf (*DO*; *dark red*) mice. WTY versus WTO, DY versus DO, WTO versus DO all *p* < 0.001 (indicated with double asterisks) and WTY versus DY *p* < 0.05 (indicated with a single asterisk) (two tailed *t*-test on arcsine transformed proportions). **d** Kernel smoothed line plots of selected enhancers overlapping hypomethylated aDMRs, ±5 kb. Replicates for WTY, DY, WTO and DO are represented by *solid blue*, *solid red*, *dashed blue* and *dashed red lines*, respectively. DMRs are highlighted in *pink* and CpGs in *black*. H3K4me1 and H3K27ac enrichment (ChIP-seq) is indicated. **e** The percentage methylation across all enhancers containing hypomethylated aDMRs unique to WT mice (6193 regions from Fig. 3b). Replicate samples (four mouse livers) are in columns and the aDMRs in rows. The intensity of the heatmap represents row scaled percentage methylation (Z-score), with values ranging from lower to higher methylation shown as *blue* to *red*. **f** Mean number of enhancer overlapping hypomethylated aDMRs per gene for WT (*blue*) and Ames dwarf (*red*) mice. Genes are split into quartiles by expression (*Q1* = highest, *Q4* = lowest). Unexpressed genes (FPKM = 0) are given (*U*). **g** Observed and expected overlap (in base pairs) of hypomethylated DMRs (WT, WT only; dwarf, dwarf only; shared, shared between WT and dwarf) with super-enhancers; **P < 0.01

Next, we set out to define the location of the hypomethylated aDMRs across the genome. First, we asked how the WT and dwarf hypomethylated aDMRs are distributed across a collection of genomic features. Although there were approximately twice as many hypomethylated aDMRs in WT mice than Ames mice (Figs. 2b and 3a), the proportionate distribution of these aDMRs across features of the genome was very similar (Fig. 3a). Most commonly, the hypomethylated aDMRs overlap genes (~60%) and introns (~50%), although they are only modestly enriched at these features, relative to the abundance of these features in the genome (Fig. 3a). Least commonly, they overlapped CpG islands (<1%) and LINEs (~5%), and were moderately depleted at these features (Fig. 3a). To further investigate, we took advantage of the many publicly available datasets for mouse liver and expanded this distribution analysis to include several ENCODE adult mouse liver histone modification ChIP-seq datasets (Fig. 3a; Additional file 5: Figure S2a; Additional file 6: Table S3; Additional file 7: Table S4). Strikingly, we observed that ~55 and ~40% of all hypomethylated aDMRs overlapped the enhancer modifications H3K4me1 and H3K27ac, with an enrichment of seven- to ninefold (*p* < 0.001; Fig. 3a). There was a more modest overlap and enrichment, or even depletion, at other histone modifications, notably the gene body modification H3K36me3 and the repressive mark H3K27me3 (Fig. 3a). In line with this enrichment at H3K4me1 and H3K27ac, there was marked overlap and enrichment at designated mouse liver enhancers, regions marked by both H3K4me1 and H3K27me3 (“Enhancers” in Fig. 3a). Of the ~47,000 identified mouse liver enhancers, 8230 and 4702 contained a hypomethylated aDMR in WT and dwarf mice, respectively (Fig. 3b), corresponding to a substantial fraction of all enhancers. Of these, a significant number (2037) were hypomethylated in both genotypes (fold enrichment of 153, *p* < 0.001; Fig. 3b). Shared hypomethylated aDMRs (Fig. 2i) are apparently generally buffered from methylation loss in the Ames mice (Fig. 3c, d). There were 6193 enhancers that contained hypomethylated aDMRs only in WT mice (Fig. 3b). These showed more marked loss of methylation in aged WT mice compared to aged dwarf mice (Fig. 3e).

Since the majority of the hypomethylated enhancers are contained within genes (Fig. 3a; Additional file 5: Figure S2b), we also assessed the relationship between enhancer hypomethylation and gene expression as determined by RNA-seq (Additional file 8: Table S5). The methylation loss per enhancer CpG was independent of expression of the gene harbouring the enhancer (Additional file 5: Figure S2c). However, hypomethylated enhancers appeared more abundant, longer and to cover a greater fraction of the gene in highly expressed genes compared to lowly expressed genes (Fig. 3f; Additional file 5: Figure S2d, e). Consistent with their high level of expression in liver, in both WT and dwarf mice the genes harbouring hypomethylated enhancers were highly enriched for liver specific genes (Additional file 5: Figure S2f). Moreover, of 30 publicly available adult mouse liver transcription factor ChIP-seq datasets (Additional file 5: Figure S2a), the factors most enriched for binding to hypomethylated aDMRs in both genotypes included key regulators of liver function (e.g. CEBPB, GR (NR3C1), RXRA, PPARA, CEBPA, HNF3A and HNF4A; *p* < 0.001; Fig. 3a). Recently, super-enhancers have been defined as clusters of enhancers that are densely bound by master transcription regulators and control expression of critical tissue-specific genes [47]. Remarkably, enhancers hypomethylated during aging were greatly enriched at such super-enhancers (Fig. 3g; Additional file 3: Table S3g). Many of these trends were exacerbated in WT mice compared to Ames dwarf mice (Fig. 3f, g; Additional file 5: Figure S2d, e). There was a significant overlap of hypomethylated genic enhancers and changes in expression of linked genes, although the vast majority of genes containing hypomethylated enhancers did not significantly alter expression (Additional file 9: Table S6). We conclude that hypomethylated aDMRs are most abundant at genes, introns and enhancers and disproportionately enriched at genic super-enhancers in highly expressed genes known to play a key role in liver function. Although the distribution of hypomethylated aDMRs is similar across the WT and dwarf epigenomes, WT mice harbour a greater number of hypomethylated genes and enhancers and the potentially disruptive effects of hypomethylation [48] are seemingly buffered by a higher level of methylation in young dwarf mice.

### Hypermethylated aDMRs are enriched at bivalent CpG islands

**Fig. 4:**
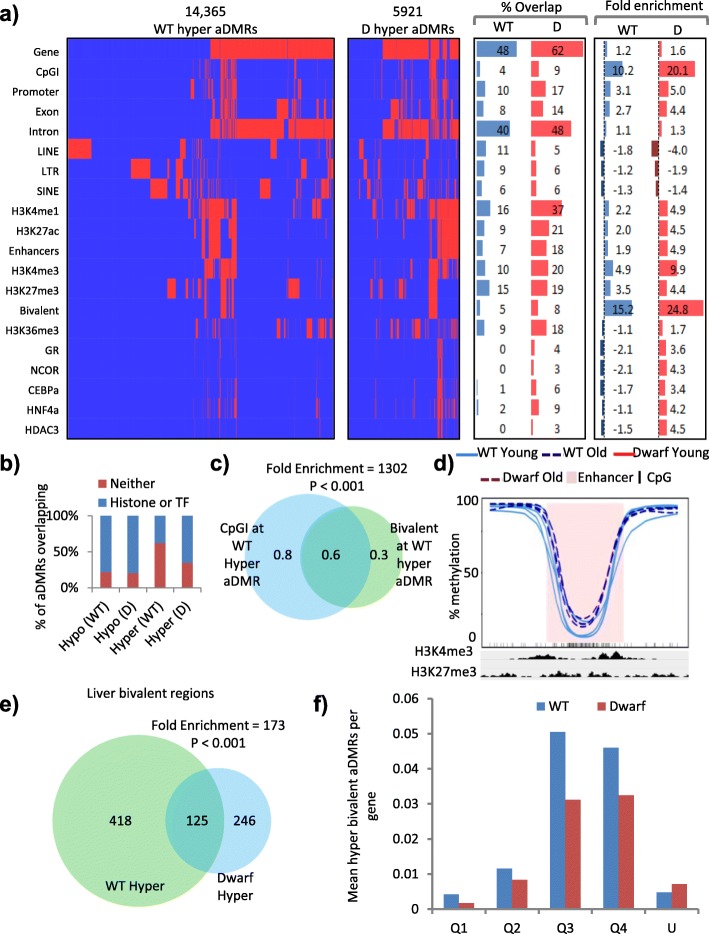
Hypermethylated aDMRs are enriched at bivalent CpG islands. **a** Clustered feature interaction maps of spatial overlap between hypermethylated aDMRs (*columns*) and a selection of genomic, histone and transcription factor features (*rows*), showing WT (*WT*; *left*) and dwarf (*D*; *centre left*) aDMRs. *Red* indicates an overlap between an aDMR and a feature and *blue* no overlap. Interaction map x-axes are scaled by number of aDMRs. The percentage overlap (*centre right*) and fold enrichment observed/expected (random) overlap (*right*; units of fold) for each feature is given. **b** Percentage of WT and Ames dwarf mice aDMRs that overlap with either histone modifications or a panel of 30 transcription factors (*Histone or TF*; *blue*) or neither (*Neither*; *red*). **c** The base pair (in mega-base pairs) overlap between hypermethylated aDMR-containing CpG islands and hypermethylated aDMR-containing bivalent regions in WT mice. Enrichment of overlap observed/expected 1302-fold, *p* < 0.001. **d** Kernel smoothed line plots of selected bivalent CpG island (*CpGI*) overlapping hypermethylated aDMRs, ±5 kb. Replicates for 2-month-old WT (*WT Young*) and 22-month-old WT (*WT Old*) mice are represented by *solid blue* and *dashed blue lines*, respectively. DMRs are highlighted in *pink* and CpGs in *black*. H3K4me3 and H3K27me3 enrichment (ChIP-seq) is indicated. **e** Liver bivalent regions that contain hypermethylated aDMRs in WT and dwarf mice. Enrichment of overlap observed/expected 173-fold, *p* < 0.001. **f** Mean number of hypermethylated bivalent aDMRs per gene for WT (*blue*) and Ames dwarf (*red*) mice. Genes are split into quartiles by expression (*Q1* = highest, *Q4* = lowest). Unexpressed genes (FPKM = 0) are given (*U*)

We then wanted to characterise and determine the location of hypermethylated aDMRs in the two genotypes of mice. Taking the same approach as for the hypomethylated aDMRs (Fig. 3a), we asked how the hypermethylated aDMRs are spatially distributed and enriched across a range of genomic features, histone modifications and transcription factors (Fig. 4a; Additional file 5: Figure S3a). Like hypomethylated aDMRs, there were more hypermethylated aDMRs in WT mice than Ames mice (Fig. 2b; Additional file 3: Table S2b). However, in contrast to hypomethylated aDMRs, the distribution of hypermethylated aDMRs appeared different between the two genotypes. This was initially apparent in a notable disparity between fold enrichment of hypermethylated aDMRs in WT and dwarf mice; at many features these aDMRs tended to show lower fold enrichment or even depletion in WT mice, particularly at transcription factor binding sites (Fig. 4a; Additional file 5: Figure S3a). Closer analysis showed that, while hypomethylated aDMRs were similarly distributed in WT and dwarf between regions marked or unmarked by histone modifications and transcription factors, hypermethylated aDMRs in WT mice were disproportionately at regions lacking histone modifications and transcription factors (Fig. 4b; Additional file 5: Figure S3b). Most of these WT-specific hypermethylated aDMRs were at regions of the genome that are relatively highly methylated even in young mice (Additional file 5: Figure S3c).

Across more richly annotated regions of the genome, hypermethylated aDMRs were distributed similarly in WT and Ames mice. In both genotypes, hypermethylated aDMRs showed the greatest overlap with genes and introns, although, like hypomethylated aDMRs, this was not enriched considering the abundance of these features in the genome (Fig. 4a). There was some enrichment of hypermethylated aDMRs at H3K27ac and H3K4me1-marked enhancers, although less so than for hypomethylated aDMRs (Figs. 3a and 4a). However, in marked contrast to hypomethylated aDMRs, hypermethylated aDMRs showed large enrichment at CpG islands and H3K4me3 and H3K27me3 marked bivalent chromatin (*p* < 0.001; Fig. 4a). There was substantial overlap between hypermethylated aDMRs at CpG islands and bivalent chromatin (fold enrichment 1302 and *p* < 0.001; Fig. 4c; Additional file 3: Table S4c), meaning that hypermethylated aDMRs were enriched at bivalent marked CpG islands (Fig. 4d). Hypermethylated bivalent regions overlapped significantly between genotypes, although there were approximately 50% more in the WT than Ames mice (Fig. 4e; Additional file 3: Table S4e). Interestingly, genes linked to these bivalent CpG islands tended to be expressed at relatively low levels (Fig. 4f), and there was no enrichment for change in expression at these genes (Additional file 9: Table S6). Gene ontology analysis showed that many of these bivalent CpG islands are linked to developmentally important genes that establish cell identity, similar to bivalent CpG islands in ES cells [49] (Additional file 5: Figure S3d). In sum, hypermethylated aDMRs are enriched at bivalent CpG islands, often of lowly expressed genes implicated in control of development and cell identity, and age-associated methylation of these islands is substantially more frequent in WT mice compared to Ames dwarf mice.

To confirm these key findings by an alternative methodology, we performed MeDIP-seq (a whole-genome sequencing-based method that isolates methylated DNA sequences using an antibody to 5-methyl-cytosine (5-mC) [50]) on a single replicate of young and old WT and Ames dwarf mice. This allowed us to plot the relative enrichment of methylated DNA reads at a set of regions, in this case the hypomethylated enhancer aDMRs and hypermethylated bivalent aDMRs that are shared between WT and Ames dwarf mice (Figs. 3b and 4e). This analysis confirmed a gain of methylation at hypermethylated bivalent regions and a decrease in methylation at hypomethylated enhancers (Additional file 3: Table S4a, b; Additional file 5: Figure S4a, b). Although this MeDIP-seq method cannot resolve methylation status at the single nucleotide level and our analysis of these data is limited to a single replicate, this alternative approach clearly validates key methylation changes in WT and Ames dwarf mice.

### Ames dwarf mice are resistant to cancer-like methylation changes during aging

**Fig. 5:**
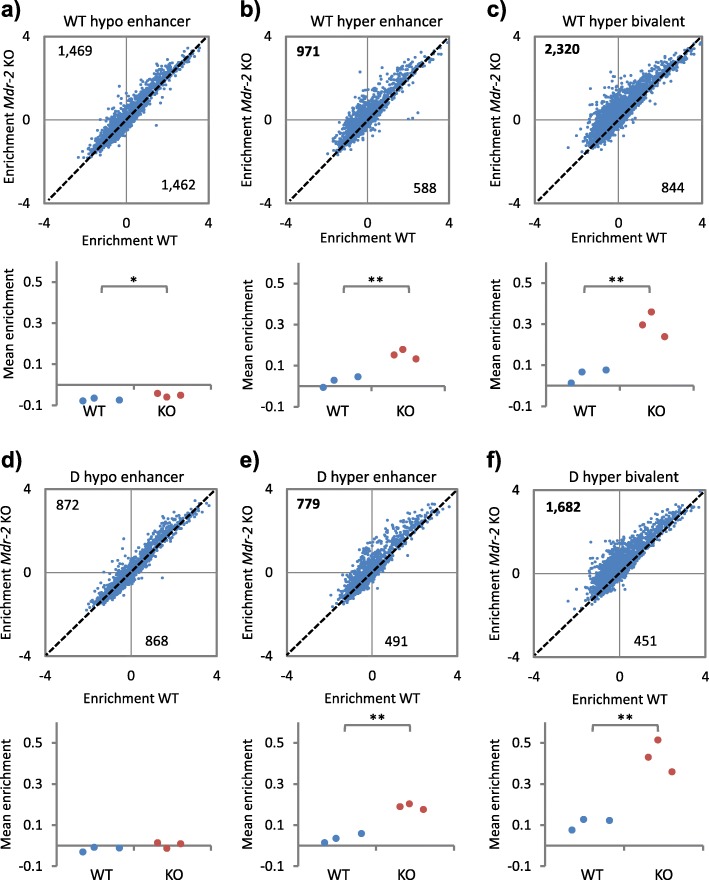
Ames dwarf mice are resistant to cancer-like methylation changes during aging. **a** Mean methylation enrichment per probe (*top panel*) at all probes within enhancers that contain WT hypomethylated aDMRs, for control (x-axis) and *Mdr-2* knockout (*KO*; y-axis) mice. *Numbers* at *top left* and *bottom right* show the number of probes above and below the *dashed diagonal*. The *bottom panel* shows the same data per mouse replicate. *P < 0.05 (two tailed *t*-test). **b** As **a** but using enhancers containing WT hypermethylated aDMRs. **P < 0.01 (two tailed *t*-test). **c** As **a** but using bivalent regions that contain WT hypermethylated aDMRs. **P < 0.01 (two tailed *t*-test). **d** As **a** but using enhancers that contain Ames dwarf hypomethylated aDMRs. *P* > 0.05 (two tailed *t*-test). **e** As **a** but using enhancers that contain Ames dwarf hypermethylated aDMRs. **P < 0.01 (two tailed *t*-test). **f** As **a** but using bivalent regions that contain Ames dwarf hypermethylated aDMRs. **P < 0.01 (two tailed *t*-test)

Bivalent CpG islands marked with H3K4me3 and H3K27me3 in ES cells tend to be DNA methylated in aged tissues and methylated and silenced in cancer [15–19], suggesting that age-associated DNA methylation can be a precursor to methylation and stable silencing in cancer. Therefore, we wanted to assess whether CpG islands methylated with age in mouse liver are also methylated on progression towards liver cancer and, if so, whether this trend was suppressed in cancer-resistant Ames dwarf mice. To do this, we analyzed DNA methylation data, obtained by methylated DNA immunoprecipitation (MeDIP) followed by array hybridization, from the late precancerous stages of HCC development in 12-month-old *Mdr2*/*Abcb4*-knockout (*Mdr2*-KO) male FVB strain mice, a well characterised model of chronic inflammation-mediated HCC [51, 52]. These mice typically exhibit chronic hepatitis from 2 months and HCC at 12–18 months. Enhancers that were hypomethylated in aged WT mice were comparably methylated in WT and *Mdr2*-KO mice (Fig. 5a; Additional file 3: Table S5a). In contrast, enhancers and bivalent CpG islands that were hypermethylated in aged WT mice also tended to be hypermethylated in *Mdr2*-KO mice (Fig. 5b, c; Additional file 3: Table S5b, c). This phenomenon was particularly marked at bivalent CpG islands. The same marked trend was also apparent at enhancers and bivalent CpG islands methylated with age in dwarf mice (Fig. 5d–f; Additional file 3: Table S5d–f). As noted previously, however, fewer such aDMRs were in dwarf compared to WT mice (Fig. 2b). These data confirm that adult liver bivalent CpG islands that are methylated during aging also tend to be methylated in pre-cancerous liver.

### Age-associated DNA methylation changes are also suppressed by calorie restriction and rapamycin

As well as genetic interventions, dietary and drug interventions also promote longevity, healthy aging and suppression of cancer. To test whether dietary and drug interventions also suppress age-associated DNA methylation changes, we examined livers from female UM-HET3 mice treated with encapsulated rapamycin (42 mg/kg of food) from 4 to 22 months’ of age and female mice on a CR diet from 4 to 22 months’ of age [53–56]. We performed WGBS-seq on DNA from whole liver, four replicates per cohort, approximately 5× coverage per sample (Additional file 4: Table S2). As controls for these UM-HET3 mice, we analyzed 2- and 22-month-old female UM-HET3 mice fed ad libitum (ad lib; Fig. 2g; Additional file 5: Figure S1a, b).

**Fig. 6:**
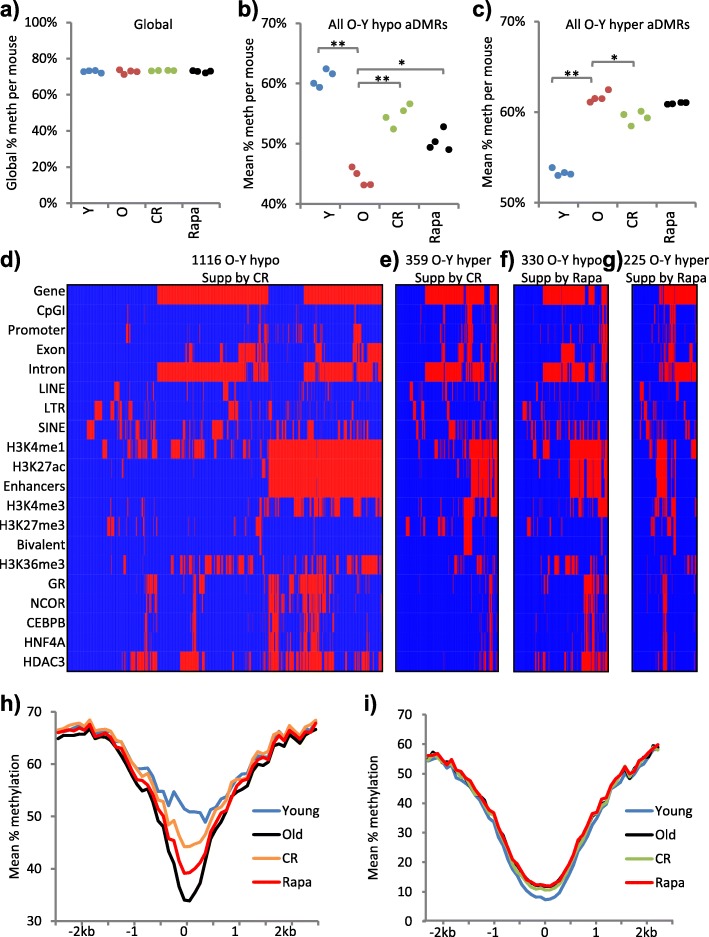
Age-associated DNA methylation changes are also suppressed by calorie restriction and rapamycin. **a** Global percentage methylation per sample for 2- (*Y*) and 22-month-old (*O*) control, 22-month-old caloric restricted (*CR*) and 22-month-old rapamycin-treated (*Rapa*) UM-HET3 mice. Part of this panel is reproduced from Additional file 5: Figure S1c. All *p* > 0.05 (two tailed *t*-test on arcsine transformed proportions). **b** Mean percentage methylation per sample across all O-Y hypomethylated aDMRs for the samples in **a**. **Y versus O, Y versus Rapa, O versus CR, all *p* < 0.001; *Y versus CR, O versus Rapa, CR versus Rapa, all *p* < 0.05 (two tailed *t*-test on arcsine transformed proportions). **c** Mean percentage methylation per sample across all O-Y hypermethylated aDMRs for the samples in **a**. **Y versus O, Y versus Rapa, Y versus CR, all *p* < 0.001; *O versus CR, CR versus Rapa *p* < 0.05, O versus Rapa *p* > 0.05 (two tailed *t*-test on arcsine transformed proportions). **d** Clustered feature interaction maps of spatial overlap between O-Y aDMRs (*columns*) and a selection of genomic, histone and transcription factor features (*rows*), showing hypomethylated aDMRs that are also CR-O hypermethylated DMRs (i.e. suppressed by CR); 1116 regions. **e** As **d** for O-Y hypermethylated aDMRs that are also CR-O hypomethylated DMRs (i.e. suppressed by CR); 359 regions. **f** As **d** for O-Y hypomethylated aDMRs that are also Rapa-O hypermethylated DMRs (i.e. suppressed by rapamycin); 330 regions. **g** As **d** for O-Y hypermethylated aDMRs that are also Rapa-O hypomethylated DMRs (i.e. suppressed by rapamycin); 225 regions. In **d**–**g**, the interaction map x-axes are scaled by number of aDMRs. **h** Composite profiles of mean percentage methylation at all hypomethylated aDMR (Old-Young, UM-HET3 mice) enhancer regions (n = 1867), showing young (*blue*), old (*black*), CR (*orange*) and rapamycin-treated (*red*). **i** Composite profiles of mean percentage methylation at hypermethylated aDMR (Old-Young) bivalent regions (n = 536), showing young (*blue*), old (*black*), CR (*green*) and rapamycin-treated (*red*)

The mean global methylation level of each cohort was very similar (72.72–73.35%) and also very similar to the mean global methylation of the Ames dwarf mice and corresponding WT (compare Fig. 1a to Fig. 6a). To begin to assess the impact of CR and rapamycin on age-associated methylation changes, we compared hypomethylated and hypermethylated aDMRs between young and old UM-HET3 mice (both fed ad lib) with the same regions in rapamycin-treated and CR old mice. At hypomethylated aDMRs, methylation loss was suppressed by CR and, to a lesser extent, by rapamycin (Fig. 6b; Additional file 3: Tables S6b and S5a–d; Additional file 5: Figure S5a–d). At hypermethylated aDMRs, methylation gain was also suppressed by CR, but not significantly by rapamycin (Fig. 6c; Additional file 3: Tables S5a, e–g; Additional file 5: S5a, e–g). At these regions, the effect of rapamycin was not significant in the whole population of hypermethylated aDMRs (Fig. 6c; Additional File 3: Table S5a; Additional file 5: Figure S5a), but was detectable in some individual aDMRs (Additional file 5: Figure S5f, g).

To assess where in the genome rapamycin and CR suppress age-associated methylation changes, we generated clustered feature interaction maps depicting the genomic distribution of regions where methylation changes were suppressed by rapamycin and CR. CR suppressed age-associated changes widely, including at genes, enhancers and CpG islands (Fig. 6d, e; Additional file 3: Table S6d, e). Rapamycin suppressed age-associated changes at a smaller number of genes, enhancers and CpG islands (Fig. 6f, g; Additional file 3: Table S6f, g). When averaged across a “composite” of all enhancers hypomethylated with age, the suppression was similarly more marked by CR than rapamycin (Fig. 6h). When averaged across all hypermethylated bivalent regions, the suppression by CR was readily apparent but modest, whereas the suppression by rapamycin was undetectable (Fig. 6i). In sum, while both CR and rapamycin suppressed age-associated hypomethylation at enhancers, at least under these protocols CR was more efficient than rapamycin. CR also suppressed hypermethylation of some bivalent regions and CpG islands, while at these regions the effect of rapamycin was detectable at a minority of regions but not in all regions combined.

In addition to suppressing age-associated methylation events, closer analysis revealed that both CR and rapamycin caused a number of hypo- and hypermethylation events that did not reflect a suppression of age-associated changes (Additional file 5: Figure S5h). However, rapamycin caused substantially more of these than did CR. These non-age-related methylation changes were widely distributed, including at genes, bivalent CpG islands and enhancers (Additional file 5: Figure S5h).

## Discussion

Here we have comprehensively mapped age-associated changes in DNA methylation across all 42 million CpGs of the genome by WGBS-seq of young and old mouse liver. In analysis and interpretation of our data, we have made extensive use of gene expression data and the many epigenomic data sets publicly available for mouse liver. Although we failed to observe global change in DNA methylation, e.g. a global hypomethylation, we did observe thousands of age-associated changes across discrete regions of the genome. The greatest number of such gains and losses of methylation occur at genes and introns, although the number of these changes is in proportion to the fraction of the genome occupied by those features. Instead, losses of methylation are most enriched at genic enhancers, including super-enhancers, within genes highly expressed in liver, and gains of methylation are most enriched at bivalent CpG islands. In sum, age-associated changes in DNA methylation are most abundant and/or enriched at various important functional and regulatory regions of the genome.

What is the cause of these age-associated changes? It is tempting to speculate that age-associated changes are linked to the dynamic nature of these regulatory and functional regions. These dynamic regions are maintained at a steady state equilibrium that may change with age. Expressed genes and enhancers are thought to be particularly dynamic regions of the epigenome [57]. More specifically, age-associated changes in DNA methylation might be linked to age-associated changes in expression of the cellular machinery that directly controls DNA methylation, such as DNMTs and TETs. Indeed, we have previously shown an age-associated increase and decrease in expression of DNMT3a and DNMT1, respectively, in mouse liver [45]. Alternatively, age-associated changes in methylation might result from changes in metabolic substrates and cofactors important for activity of DNMTs and TETs, such as S-adenosylmethionine (SAM) and α-ketoglutarate, respectively [58]. However, there must also be additional sequence and/or epigenetic determinants of methylation gains and losses to explain why some regions, such as bivalent CpG islands, gain methylation whereas others, such as enhancers, lose methylation with age.

What is the consequence of these age-associated changes in methylation? Losses of methylation at enhancers are only weakly linked to changes in expression of linked genes, and some genes increase and others decrease in expression. Recent studies suggested that DNA methylation of enhancers is required for their functional integrity [48, 59]. So, while age-associated loss of methylation at enhancers in these moderately old mice (22 months old) is only modestly linked to changes in gene expression, it is conceivable that this methylation loss is a precursor to more dramatic changes in methylation and expression in very old mice or perhaps after tissue stress. In contrast to enhancers, gains of DNA methylation at bivalent CpG islands are not enriched for changes in gene expression. Most of these genes are expressed at comparatively low levels even in normal young tissue, and a gain of methylation at the promoter CpG island is not expected to increase their expression. Importantly, however, age-associated changes at bivalent CpG islands are linked to hypermethylation in cancer, suggesting that age-associated gain in methylation can be a precursor to cancer, for example by blocking activation of pro-differentiation and development genes, as proposed previously [15, 18].

Age-associated changes in DNA methylation are suppressed by genetic, dietary and drug interventions that extend lifespan and delay/suppress the incidence of cancer, specifically the *Prop1* mutation in the Ames dwarf mouse, CR and rapamycin [1, 5]. Each of these interventions suppresses age-associated changes in methylation at genes, enhancers and bivalent CpG islands. Consistent with the aforementioned proposal that age-associated methylation changes are linked to control of the DNA methylation machinery and/or its metabolic regulators, Ames dwarf mice do display atypical methionine metabolism, methionine being a source of the SAM that is required for DNA methylation. Components of this amino acid pathway are upregulated in Ames mice, leading to higher enzyme activities, including of glycine N-methyltransferase (GNMT), an enzyme that converts SAM to S-adenosyl-homocysteine and sarcosine. Moreover, the methyltransferase enzymes important in DNA methylation and methionine metabolism are affected by the presence or absence of GH. Methionine flux assays confirm the enhanced enzyme activities, demonstrating that transmethylation and transsulphuration are markedly elevated in dwarf mice [60, 61]. Thus, elevated GNMT in Ames dwarf mice might depress age-associated methylation of CpG islands, perhaps contributing to delayed cancer incidence [2, 3]. Similarly, CR and rapamycin might suppress the incidence of cancer [5–7], at least in part, by suppressing methylation of bivalent CpG island promoters. Aside from cancer suppression, the other shared benefits of these genetic, dietary and drug interventions for maintenance of tissue and systemic function into old age might depend on suppression of super-enhancer hypomethylation and so preservation of tissue specific enhancer integrity, gene expression programs and tissue function [48]. Typically, the effect of CR on the epigenome was greater than rapamycin, in line with the greater extension of lifespan by CR than rapamycin, at least under the protocols tested here [55, 56].

Notwithstanding the more efficient suppression of age-associated epigenetic changes by CR, the epigenetic effects of CR and rapamycin were not identical and this might further underlie some of the differences between them that have been noted in previous studies, for example in endocrine and metabolic phenotypes and gene expression profiles [55, 62]. Of note, rapamycin in particular appears to induce additional changes unrelated to age-associated changes. While both CR and rapamycin induced these non-age-related effects, this effect was much more marked for rapamycin. These non age-related epigenetic changes include gains of methylation at genes, enhancers and CpG islands and losses of methylation at genes and enhancers. Conceivably, such non age-related effects of rapamycin in liver and other tissues may contribute to at least some of the well-documented harmful side effects of rapamycin, such as glucose intolerance, increased incidence of testicular degeneration and cataracts [54, 55]. Detrimental effects of rapamycin-like drugs, including dyslipidemia, hyperlipidemia and risk of diabetes, have also been noted in humans [63, 64]. Of course, such adverse consequences of rapamycin might also have a non-epigenetic basis. Regardless, this study is a first comparison of the effect of diverse genetic, dietary and drug interventions on the epigenetic landscape and a foundation for understanding their influence on epigenetic determinants of chronological and biological aging.

## Conclusions

We conclude that aging of mouse liver is associated with marked DNA methylation changes to critical gene regulatory sequences, including gene promoters and enhancers of highly expressed genes. Distinct longevity-promoting interventions, specifically genetic, dietary and drug interventions, suppress some age-associated methylation changes, consistent with the idea that these interventions exert their beneficial effects, in part, by modulation of the epigenome. Together with the accompanying paper [65], our studies suggest that hypomethylation of genic enhancers may constitute a biological age clock that can predict liver function after stress.

## Additional files

Additional file 1: Supplementary dataset 1. WGBS alignment statistics for the Ames and WT data. (XLSX 11 kb)

Additional file 2: Supplementary methods. Additional methods for this manuscript. (DOCX 38 kb)

Additional file 3: Supplementary statistical data. The results and description of statistical methods used in each figure. (XLSX 30 kb)

Additional file 4: Supplementary dataset 2. WGBS alignment statistics for the UM-HET3 data. (XLSX 11 kb)

Additional file 5: Supplementary figures. Supplementary figures for this manuscript. (PDF 2707 kb)

Additional file 6: Supplementary dataset 3. Accession codes and details of publically available histone datasets used in this analysis. (XLSX 8 kb)

Additional file 7: Supplementary dataset 4. Accession codes and details of publically available transcription factor datasets used in this analysis. (XLSX 10 kb)

Additional file 8: Supplementary dataset 5. Alignment statistics for the Ames RNA-seq experiment. (XLSX 11 kb)

Additional file 9: Supplementary dataset 6. RNA-seq results. (XLSX 9 kb)
