# Supplementary material for: Diverse interventions that extend mouse lifespan suppress shared age-associated epigenetic changes at critical gene regulatory regions
Source: Genome Biol. 2017 Mar 28;18:58. doi: 10.1186/s13059-017-1185-3 (PMC5370462; doi:10.1186/s13059-017-1185-3)
Supplement: Supplementary file 2 — Supplementary methods. Additional methods for this manuscript. (DOCX 38 kb) [file 13059_2017_1185_MOESM2_ESM.docx]

**Methods**

*Mice.* Ames dwarf mice were derived from a closed colony with a heterogeneous background (over 25 years) at the University of North Dakota [1]. Dwarf mice were generated by mating either homozygous (df/df) or heterozygous (df/+) dwarf males with carrier females (df/+).  All mice were bred and maintained at the University of North Dakota Center for Biomedical Research under controlled conditions of photoperiod (12:12 h light/dark cycle) and temperature (22 -/+1 °C) and *ad libitum* access to food (Teklad #8640) and water. Animal procedures were reviewed and approved by the UND Institutional Animal Care and Use Committee. Liver tissue from male WT and Ames dwarf mice was collected at 2 and 22 months of age (n=4/genotype/age). Liver DNA was isolated (Qiagen).

Genetically heterogeneous UM-HET3 female mice were bred and housed at

the University of Michigan.  They are the offspring of (BALB/cByJ x C57BL/6J)F1 mothers and (C3H/HeJ x DBA/2J)F1 fathers.  They were housed in a specific-pathogen free colony at four mice/cage and given a diet based on Purina 5LG6.  Some mice were given encapsulated rapamycin at a drug dose of 42 ppm from 4 months of age [2].  For other mice, a calorie restricted diet was initiated at 4 months; these mice received 60% of the food consumed by age-matched controls, after a two week run-in period at 80% [3].

**Whole-genome Bisulfite sequencing**

*Carried out by BGI, Shenzhen.* Genomic DNA was isolated from mice livers using DNeasy blood and tissue kit (Quiagen) according to the manufacturers’ protocol. Whole genome bisulfite sequencing of mouse DNA was performed by the Beijing Genomics Institute (BGI, Shenzhen, China). Briefly, DNA was subjected to QC check by agarose gel electrophoresis and quantified using qubit (Invitrogen). Almost 15ug of DNA was sent for bisulfite sequencing. After arrival and qualifying the samples, DNA was fragmented by sonication to a mean size of approximately 100-300 bp, followed by DNA-end repair, the addition of dA to 3’-end and, finally, ligation of the methylated-sequencing adapter according to the BGI’s standard protocol. The bisulfite conversion of the adaptor-added DNA was carried out using ZYMO EZ DNA Methylation-Gold kit (Zymo Research). Bisulfite converted DNA was subjected to 90bp paired-end sequencing using an Illumina HiSeq-4000 machine (Illumina, San Diego, California).

**Data analysis/statistics**

*Pre-processing of bisulfite sequencing reads.*  Sequenced reads were assessed for quality using FastQC (version 0.10.0) prior to having all adapters and low quality sequence tails removed using trim-galore (version 0.3.0).

*Alignment of bisulfite sequencing reads.* Sequence reads are transformed *in silico* to fully bisulfite-converted forward (C→T) and reverse (G→A) reads. The converted sequences are aligned against a converted UCSC (mm9) genome in each combination: (1) forward (C→T) reads align to forward (C→T) genome; (2) reverse (G→A) reads align to reverse (G→A) genome; (3) forward (C→T) reads align to reverse (G→A) genome; (4) reverse (G→A) reads align to forward (C→T) genome. During the library preparation process [4], genomic fragments representing alignments (3) and (4) are generated in the PCR step; however, they are not sequenced and only fragments corresponding to alignments (1) and (2) are retained. Alignment was carried out using Bismark [5] (version 0.10.1), based on the Bowtie2 [4] aligner (version 2.1.0) on the UCSC mm9 mouse genome.

For each aligned sequence tag, the original unconverted sequence is compared against the original unconverted reference genome and the methylation status is inferred. Sequences aligned from (1) and (2) give information on cytosines on the forward and reverse strands respectively.

To remove PCR bias, a deduplication step removes potential duplicate reads, where both ends of the fragment align to the same genomic positions on the same strand, only one of these reads is retained. To control for potential incomplete bisulfite treatment, any reads with more than three methylated cytosines in non-CpG contexts are discarded. Supplementary Dataset 1 and Dataset 3 details the sequence yields at each stage of this process for the young and old Ames and WT control data-set and the caloric restriction and rapamycin data-set respectively.

*Identification of methylated cytosines.* Processed reads are aggregated on a per CpG basis (number of bases read supporting methylated/unmethylated status). At this stage we collapse CpG dyads using a bespoke script that combines methylated and unmethylated coverage scores for each CpG dyad into a single score for the cytosine on the forward strand, thus artificially increasing the coverage with a slight loss in spatial resolution. A two-tailed Fisher exact test was used to identify CpGs that were differentially methylated. Only CpGs with at least ten reads within each comparable condition were considered for testing. P-values were corrected using the Benjimini-Hochberg (BH-) FDR function to control false positives at a rate of 5%.

*Identification of DMRs.* DMRs were calculated using a sliding window approach, window size 500 bp. Windows start at an individual CpG and extend 500 bp, ending at the final CpG contained within each window. At each window a two-tailed Fisher exact test was performed to determine DMR significance, alongside a chi-squared test of heterogeneity across the 4 mouse replicates within each cohort. Both tests were conducted at high precision using the Python arbitrary precision mathematics (mpmath) package. Both chi-squared and Fisher exact test p-values were multi-sample corrected using BH-FDR. The corrected Fisher exact test p-value is used to determine significant difference within each window while the corrected chi-squared is used to measure heterogeneity within each cohort. DMRs were selected by a BH-FDR corrected p-value less than 0.05 with each measured cohort displaying non-significant heterogeneity via the chi-squared test (> 0.05). At this stage replicates were pooled for subsequent analysis.

*Percentage in genomic windows.* The percentage CpG methylation for any given window was calculated as the total number of cytosines sequence (at CpG sites for that window), divided by the total number of methylated and unmethylated cytosines (at CpG sites for that window), multiplied by 100.

*Difference and relative difference in CpG methylation.* Difference in CpG methylation was defined as the difference between treated and (relative) control percentage methylation. Relative difference in CpG methylation was defined as the difference in CpG methylation divided by the control percentage methylation.

*Global methylation.* A genome wide methylation level was determined by totalling all methylated and dividing by the total methylated and unmethylated counts in the entire data-set for each replicate.

*Determination of overlaps.* Overlaps were computed on a per base pair basis between two datasets (A and B). For every region within A the number of base pairs that were occupied by a region within B was computed. A permutation test was carried out to determine the background genomic average expected overlap. 1000 sets of regions with properties (length distribution and chromosome distribution) equal to those of set B were generated. Randomly generated regions of B were prevented from being generated within unsequenced regions of the genome (as defined by UCSC mapping and sequencing track—gap). The overlap of A and B was repeated for each randomly generated set of B to determine the average expected random overlap. P-values were estimated empirically from the observed overlaps of the randomly generated sets.

*Composite methylation profiling.* The midpoints of a series of regions of interest were taken and used as a base.  The area around the midpoint of each feature was then split into 100bp windows spanning 2.5kb upstream and downstream of this central position.  The average methylation proportion was then calculated for each window for every feature.  A global mean was then taken for each window across all features to aggregate a composite of the mean methylation per window across all probed features.

*Clustered feature interaction maps.* We created the heatmaps using a bespoke script that accepts a set of DMRs as well as a selection of assorted features and creates a binary matrix – 1 for interaction (if the two features overlap by more than 10bp), 0 for none. The matrix is then clustered in R using Wards method on the axis that represents our DMR set.

**ChIP-seq**

For analysis ChIP-seq single-end reads were trimmed using trim-galore (v0.3.0) and aligned to the mouse genome (mm9) using the Bowtie2 alignment software removing reads with quality < 15. Non-unique and duplicate reads were removed using samtools and Picard tools (v1.98) respectively.

*Histone mark peak calling.* Regions of histone occupancy were determined using SICER (v1.1) [6] using: a redundancy threshold of 1, window size of 200bp, fragment size of 150, effective genome fraction of 0.75, gap size of 200 and R of 0.01. When using public data the relevant input controls were used (Supplementary Dataset 4). Where multiple replicates existed, bedtools [7] was used to intersect called peaks, thus generating a set of common consensus regions.

*Transcription factor peak calling.* Regions of ChIP enrichment were determined using MACS (v1.4) – which empirically models the fragment length of ChIP reads and calling significant peaks with p-values ≤ 0.00005 (1E-5) [8]. Datasets listed in Supplementary Dataset 5.

**RNA-seq**

Paired-end reads were aligned to the mouse genome (mm9) using a splicing-aware aligner (tophat2) [9]. Only unique reads were retained. See Supplementary Table 1. Reference splice junctions were provided by a reference transcriptome (Ensembl build 67), and novel splicing junctions determined by detecting reads that spanned exons that were not in the reference annotation. True read abundance at each transcript isoform was assessed using HTSeq (Python) before determining differential expression with the tool DESeq2 [10] which models mean-variance dependence within the sample set. Significance was determined using an FDR corrected p-value <= 0.05. Alignment statistics for the Ames dwarf and relative WT control RNA-seq are shown in Supplementary Dataset 6.

**Visualisations**

*Heatmaps and clustered feature interaction maps.* Maps were created in R using the *ggplots* package. Those heatmaps displaying normalised methylation (Z-score) across a series of replicates were clustered using a Pearson correlative clustering approach whereas clustered feature interaction maps outlining the intersection of DMRs and transcription factor, histone and annotated genomic regions were clustered along the x-axis using Ward’s hierarchical clustering method. Features on y-axis were manually ordered.

*Smoothed methylation plots.* The pooled whole genome methylation data or data for each replicate were processed using the BSmooth algorithm from the bsseq (v0.8.0) package within Bioconductor as described in [11]. Methylation levels were kernel smoothed and plotted against a range of DMR and annotated features in bed format.

*PCA.* Principle component analysis was performed in R using the *prcomp* method.

*UCSC Traces.* Aggregate methylation files were converted to crude percentile coverage files in the bedGraph format, before being converted to bigwig using UCSC tools.

**Validation**

Genomic DNA was extracted from frozen ground-up liver samples and fragmented to a range between 100bp to 400bp (mean 217bp) in size using a Covaris sonicator prior to immunoprecipitation with 5mC antibody (Eurogentic #BI-MECY-1000). For full DNA and HmeDIP and MeDIP protocols see Thomson et al 2015 - Scientific Reports [12]. In brief 5mC marked DNA fragments were enriched following antibody enrichment to magnetic M-280 Sheep anti-mouse IgG Dynabeads (Thermo Fisher) and purified using DNA Clean and Concentrator™ (Zymo Research) prior to preparation for genome wide sequencing on the Ion Proton semiconductor sequencer. Sequencing reads were aligned to the mouse genome mm9 reference using TMAP software and 5mC values calculated by binning read counts across 150bp windows throughout the genome. Resulting datasets were then normalised by total read count prior to subtraction of background input signals.

Average normalised MeDIP signals were calculated across bivalent and enhancer regions selected for having overlapping DMRs in both the WT Ames control and the dwarf based on loci described in the WGBS data. The level of 5mC across each of these sets of loci was then plotted as a boxplot.

**Availability of Data**

Our data is freely available under GEO accession number GSE89275.

**External Data**

A list of the public data and the GEO accession numbers are contained in Supplementary Datasets 4 and 5 for the histone mark and transcription factor data respectively.

**References**

1. Brown-Borg, H.M., et al., *Growth hormone signaling is necessary for lifespan extension by dietary methionine.* Aging Cell, 2014. **13**(6): p. 1019-27.

2. Wilkinson, J.E., et al., *Rapamycin slows aging in mice.* Aging Cell, 2012. **11**(4): p. 675-82.

3. Harrison, D.E., et al., *Acarbose, 17-alpha-estradiol, and nordihydroguaiaretic acid extend mouse lifespan preferentially in males.* Aging Cell, 2014. **13**(2): p. 273-82.

4. Langmead, B., et al., *Ultrafast and memory-efficient alignment of short DNA sequences to the human genome.* Genome Biol, 2009. **10**(3): p. R25.

5. Krueger, F. and S.R. Andrews, *Bismark: a flexible aligner and methylation caller for Bisulfite-Seq applications.* Bioinformatics, 2011. **27**(11): p. 1571-2.

6. Zang, C., et al., *A clustering approach for identification of enriched domains from histone modification ChIP-Seq data.* Bioinformatics, 2009. **25**(15): p. 1952-8.

7. Quinlan, A.R. and I.M. Hall, *BEDTools: a flexible suite of utilities for comparing genomic features.* Bioinformatics, 2010. **26**(6): p. 841-2.

8. Zhang, Y., et al., *Model-based analysis of ChIP-Seq (MACS).* Genome Biol, 2008. **9**(9): p. R137.

9. Kim, D., et al., *TopHat2: accurate alignment of transcriptomes in the presence of insertions, deletions and gene fusions.* Genome Biol, 2013. **14**(4): p. R36.

10. Love, M.I., W. Huber, and S. Anders, *Moderated estimation of fold change and dispersion for RNA-seq data with DESeq2.* Genome Biol, 2014. **15**(12): p. 550.

11. Hansen, K.D., B. Langmead, and R.A. Irizarry, *BSmooth: from whole genome bisulfite sequencing reads to differentially methylated regions.* Genome Biol, 2012. **13**(10): p. R83.

12. Thomson, John P. et al., DNA immunoprecipitation semiconductor sequencing (DIP-SC-seq) as a rapid method to generate genome wide epigenetic signatures., Scientific Reports, 2015
