# Supplementary material for: Diverse interventions that extend mouse lifespan suppress shared age-associated epigenetic changes at critical gene regulatory regions
Source: Genome Biol. 2017 Mar 28;18:58. doi: 10.1186/s13059-017-1185-3 (PMC5370462; doi:10.1186/s13059-017-1185-3)
Supplement: Supplementary file 5 — Supplementary figures. Supplementary figures for this manuscript. (PDF 2707 kb) [file 13059_2017_1185_MOESM5_ESM.pdf]

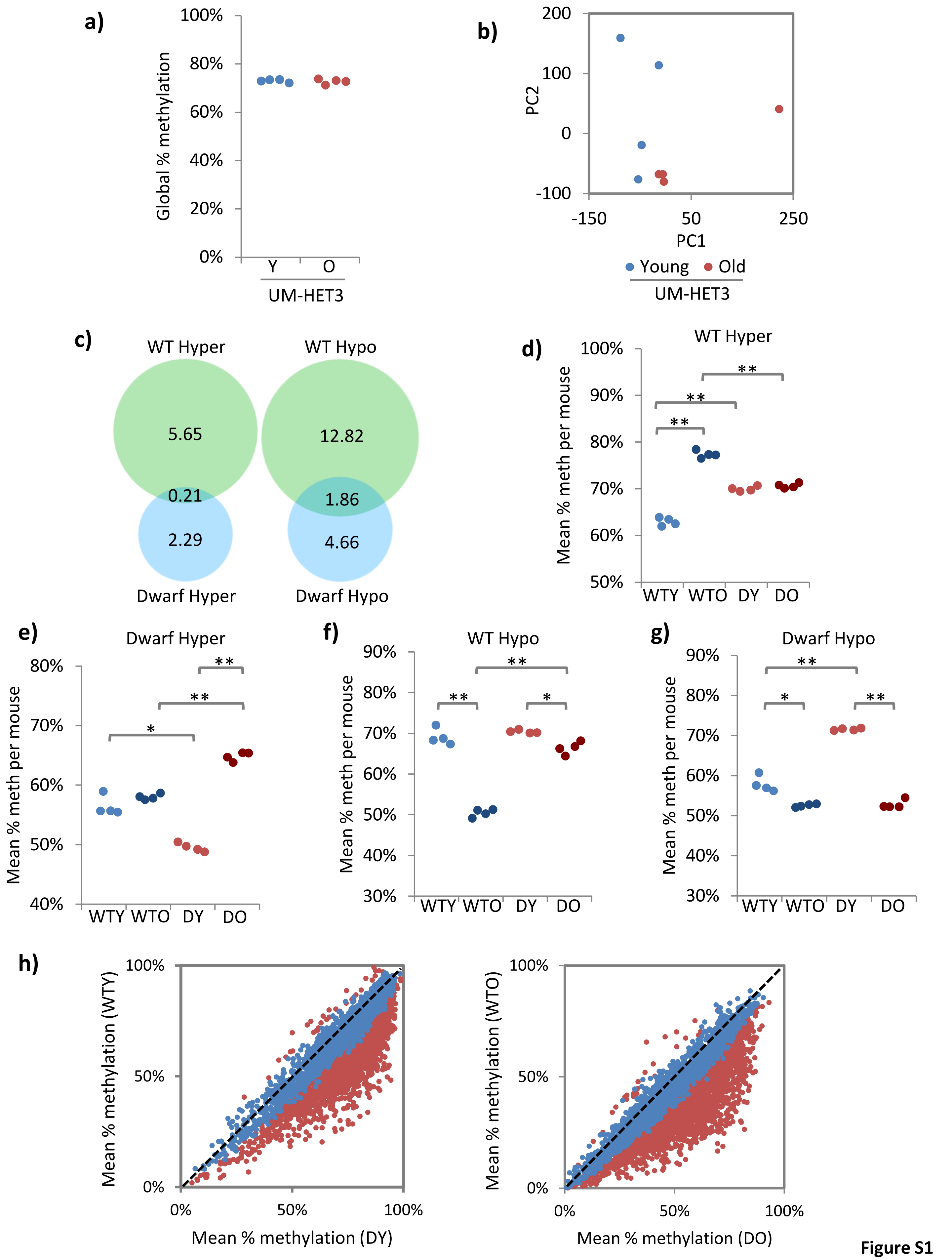

a)
Global % methylation
100%
80%
60%
40%
20%
0%
Y
O
UM-HET3
b)
PC2
200
100
0
-100
-150
50
250
PC1
Young
Old
UM-HET3
c)
WT Hyper
WT Hypo
5.65
12.82
0.21
1.86
2.29
4.66
Dwarf Hyper
Dwarf Hypo
d)
WT Hyper
Mean % meth per mouse
100%
90%
80%
70%
60%
50%
WTY
WTO
DY
DO
e)
Dwarf Hyper
80%
70%
60%
50%
40%
f)
WT Hypo
90%
30%
g)
Dwarf Hypo
h)
Mean % methylation (WTY)
Mean % methylation (DY)
Mean % methylation (WTO)
Mean % methylation (DO)
0%
50%
100%

Figure S1

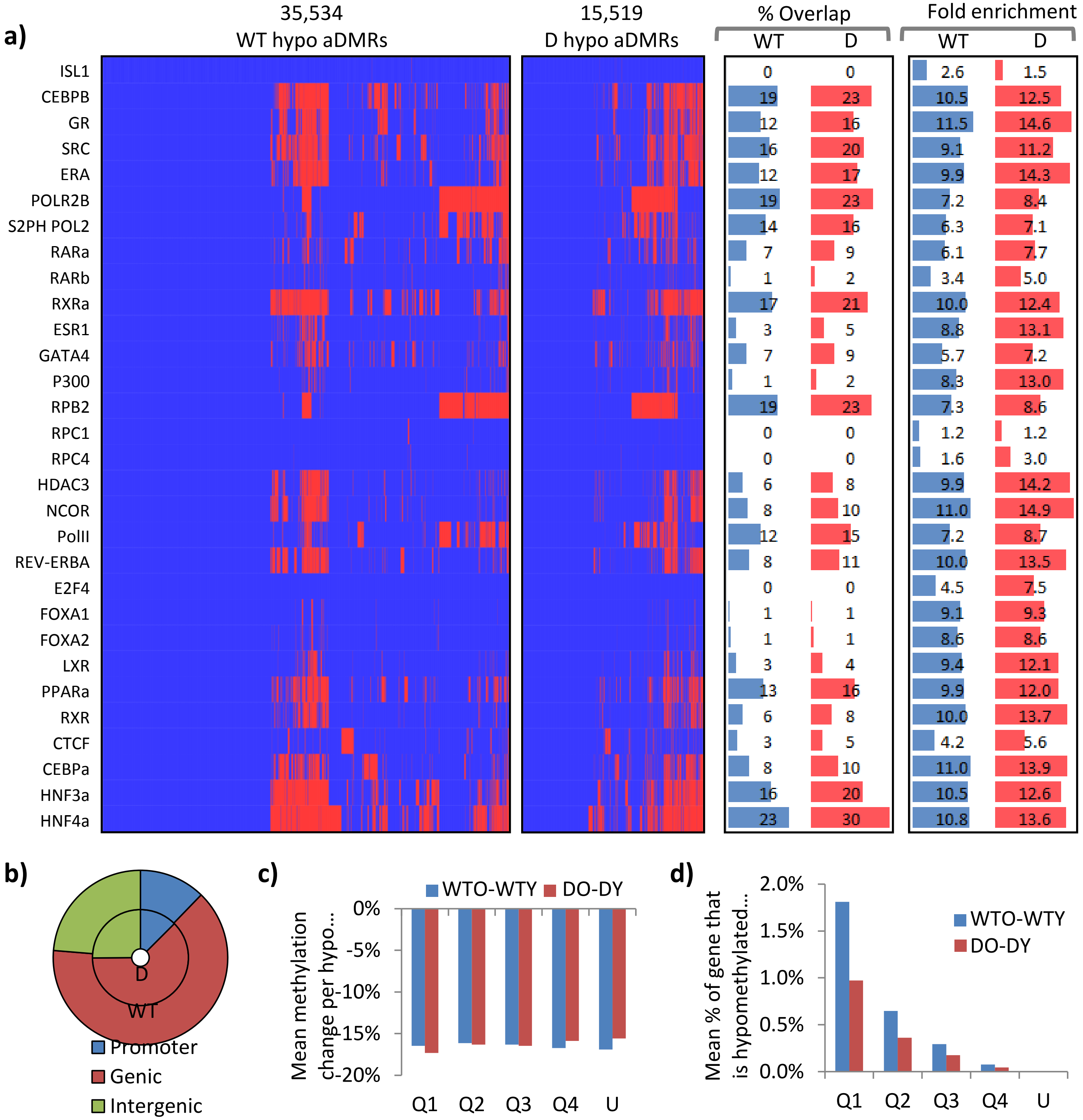

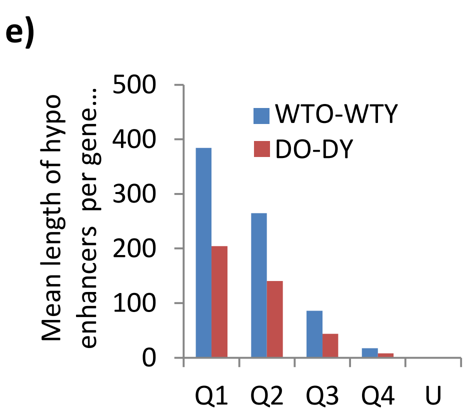

f)

	Term	Count	%	Fold Enrich	FDR
WT	Liver	1300	33.74	2.00	2.4551E-181
	Kidney	630	16.35	1.53	5.90611E-31
	Bone marrow	397	10.30	1.48	4.72221E-15
	Mammary tumor	692	17.96	1.38	6.60656E-20
D	*Liver*	*844*	*34.56*	*2.05*	*5.8134E-113*
	Bone marrow	*279*	*11.43*	*1.64*	*5.25917E-15*
	Kidney	*391*	*16.01*	*1.50*	*2.06576E-15*
	Mammary tumor	*450*	*18.43*	*1.42*	*4.32987E-13*

Figure S2

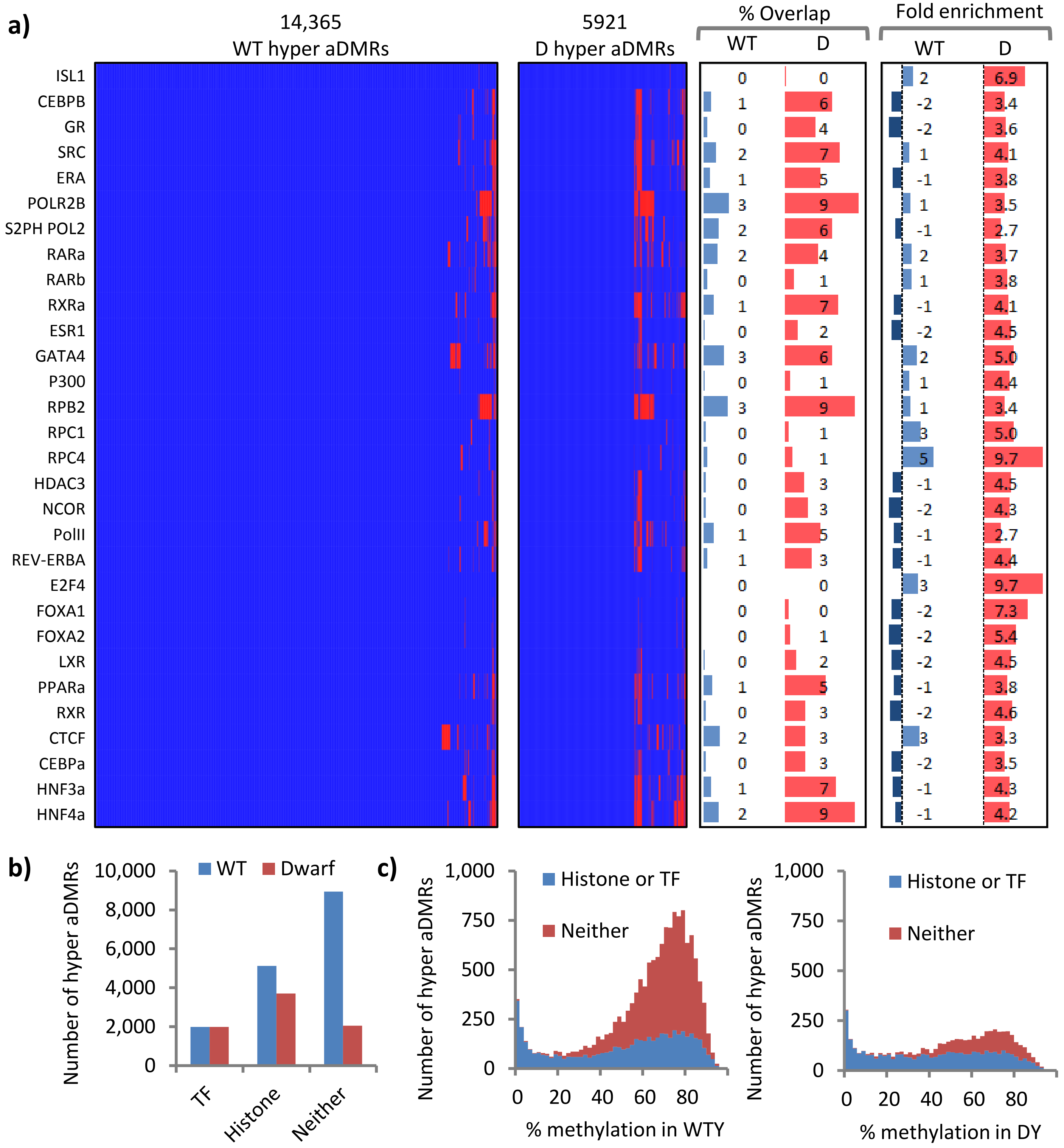

d)

WT hypermethylated bivalent regions

Top molecular functions	p-value range
Cellular Development	$3.8x10^{-4}$ - $7.3x10^{-26}$
Cellular Assembly and Organization	$3.7x10^{-4}$ - $2.6x10^{-16}$
Cellular Function and Maintenance	$3.7x10^{-4}$ - $2.6x10^{-16}$
Gene Expression	$2.9x10^{-4}$ - $7.4x10^{-14}$
Cellular Growth and Proliferation	$3.6x10^{-4}$ - $2.6x10^{-13}$

Dwarf hypermethylated bivalent regions

Top molecular functions	p-value range
Cellular Development	$7.8x10^{-4}$ - $1.2x10^{-17}$
Gene Expression	$4.6x10^{-9}$ - $3.3x10^{-10}$
Cellular Movement	$8.5x10^{-4}$ - $5.3x10^{-9}$
Cellular Growth and Proliferation	$7.8x10^{-4}$ - $5.7x10^{-9}$
Cell Morphology	$8.4x10^{-4}$ - $3.8x10^{-8}$

Figure S3

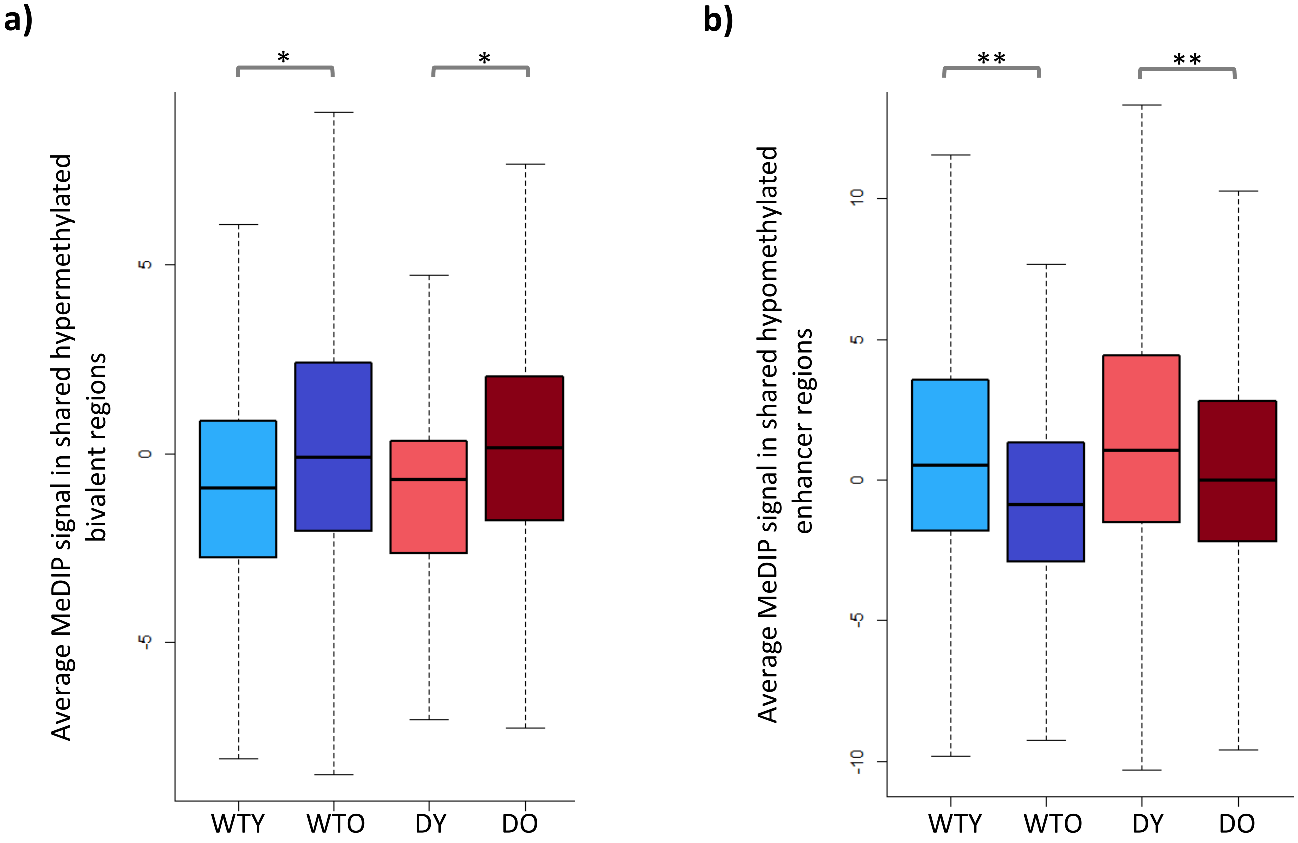
a)
b)
*
*
**
**
Average MeDIP signal in shared hypermethylated bivalent regions
Average MeDIP signal in shared hypomethylated enhancer regions
5
0
-5
10
5
0
-5
-10
WTY
WTO
DY
DO
WTY
WTO
DY
DO

Figure S4

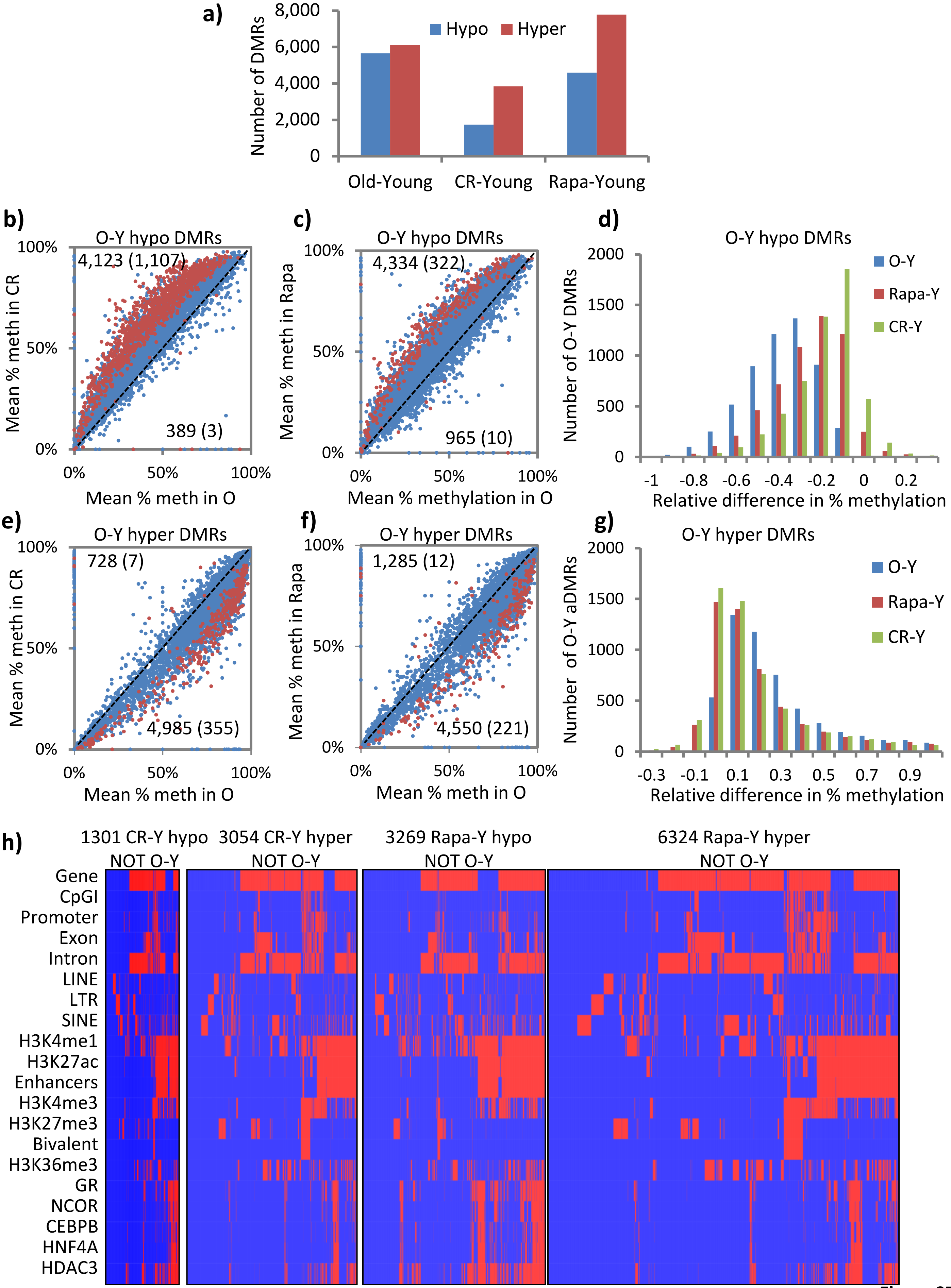

a)
Number of DMRs
8,000
6,000
4,000
2,000
0
Hypo
Hyper
Old-Young
CR-Young
Rapa-Young
b)
O-Y hypo DMRs
4,123 (1,107)
389 (3)
Mean % meth in CR
Mean % meth in O
c)
O-Y hypo DMRs
4,334 (322)
965 (10)
Mean % meth in Rapa
Mean % methylation in O
d)
O-Y hypo DMRs
Number of O-Y DMRs
O-Y
Rapa-Y
CR-Y
Relative difference in % methylation
e)
O-Y hyper DMRs
728 (7)
4,985 (355)
Mean % meth in CR
Mean % meth in O
f)
O-Y hyper DMRs
1,285 (12)
4,550 (221)
Mean % meth in Rapa
Mean % meth in O
g)
O-Y hyper DMRs
Number of O-Y aDMRs
O-Y
Rapa-Y
CR-Y
Relative difference in % methylation
h)
1301 CR-Y hypo NOT O-Y
3054 CR-Y hyper NOT O-Y
3269 Rapa-Y hypo NOT O-Y
6324 Rapa-Y hyper NOT O-Y
Gene
CpGI
Promoter
Exon
Intron
LINE
LTR
SINE
H3K4me1
H3K27ac
Enhancers
H3K4me3
H3K27me3
Bivalent
H3K36me3
GR
NCOR
CEBPB
HNF4A
HDAC3

Figure S5

Figure S1. Supplementary data to Figure 2. a) Global % methylation per sample, for 2 month (Y), 22 month (O) UM-HET3 mice. P>0.05 (two tailed t-test on arcsine transformed proportions). **b)** Principal component analysis of Bisulphite sequencing % methylation, for Y and O UM-HET3 mice. **c)** Venn diagrams of overlap in mega bp between WT and dwarf hypermethylated aDMRs (left) and hypomethylated aDMRs (right). Both WT and dwarf, P < 0.001. **d)** Mean % methylation per sample across all hypermethylated aDMRs that present in WT but not dwarf mice. WTY vs WTO, WTY vs DY, WTO vs DO P<0.001 indicated by ** and DY vs DO P>0.05 (two tailed t-test on arcsine transformed proportions). **e)** Mean % methylation per sample across all hypermethylated aDMRs that present in dwarf but not WT mice. DY vs DO, WTO vs DO P<0.001 indicated by **, WTY vs DY P<0.05 indicated by *, WTY vs WTO P>0.05 (two tailed t-test on arcsine transformed proportions). **f)** Mean % methylation per sample across all hypomethylated aDMRs that present in WT but not dwarf mice. WTY vs WTO, WTO vs DO P<0.001 indicated by **, DY vs DO P<0.05 indicated by *, WTY vs DY P>0.05 (two tailed t-test on arcsine transformed proportions). **g)** Mean % methylation per sample across all hypomethylated aDMRs that present in dwarf but not WT mice. DY vs DO, WTY vs DY P<0.001 indicated by **, WTY vs WTO P<0.05 indicated by *, WTO vs DO P>0.05 (two tailed t-test on arcsine transformed proportions). **h)** Left panel : Scatter plot of all hypomethylated aDMRs common to both WT and Ames dwarf mice, showing mean % methylation (per region) in 2 month old WT mice (y-axis) and 2 month old dwarf mice (x-axis). Regions that are significantly different in % methylation between WT and dwarf are shown in red. Right panel : As left, but in old mice.

Figure S2: Supplementary data to Figure 3. a) Clustered feature interaction maps of spatial overlap between hypomethylated aDMRs (columns) and a panel of 30 transcription factors (rows), showing WT (WT)(left) and dwarf (D)(center left) aDMRs. Red indicates an overlap between aDMR and feature and blue no overlap. Interaction map x axes are scaled by number of aDMRs. The % overlap (center right) and fold enrichment observed/expected (random) overlap (right)(units of

fold) for each feature is given. **b)** Proportion of enhancers overlapping WT (outer ring) and dwarf (inner ring) hypomethylated aDMRs that are: promoter (blue), genic (and not promoter)(red) and intergenic (and not promoter)(green). **c)** Mean difference in % methylation (22 month (O) – 2 month (Y)) per CpG within hypomethylated aDMRs that overlap genic enhancers. CpGs are split into quartiles by expression of the nearest gene (Q1 = highest, Q4 lowest). Unexpressed genes (FPKM = 0) are given (U). WT (blue) and dwarf (red) values are shown. **d)** Mean percent of gene that is a hypomethylated aDMR that overlaps genic enhancers for WT (Blue) and Ames dwarf (Red). Genes are split into quartiles by expression (Q1 = highest, Q4 = lowest). Unexpressed genes (FPKM = 0) are given (U). WT (blue) and dwarf (red) values are shown. **e)** Mean size in bp that is a hypomethylated aDMR that overlaps genic enhancers for WT (Blue) and Ames dwarf (Red). Genes are split into quartiles by expression (Q1 = highest, Q4 lowest). Unexpressed genes (FPKM = 0) are given (U). WT (blue) and dwarf (red) values are shown. **f)** Most highly enriched tissue expression profiles (David, UP tissue) amongst all WT (top table) and dwarf (bottom table, italicized) genes with aDMR overlapping enhancers.

Figure S3. Supplementary data to Figure 4. a) Clustered feature interaction maps of spatial overlap between hypermethylated aDMRs (columns) and a panel of 30 transcription factors (rows), showing WT (WT)(left) and dwarf (D)(center left) aDMRs. Red indicates an overlap between aDMR and feature and blue no overlap. Interaction map x axes are scaled by number of aDMRs. The % overlap (center right) and fold enrichment observed/expected (random) overlap (right)(units of fold) for each feature is given. **b)** Barchart of the number of WT (blue) and Ames dwarf (red) aDMRs that overlap with either histone modifications or panel of 30 transcription factors (TF and Histone) or neither (Neither). **c)** Histograms of number of hypermethylated aDMRs (y-axis) by % methylation (x axis) in 2 month WT (left) and Ames dwarf (right) mice. Showing aDMRs that overlap with either histone modifications or panel of 30 transcription factors (blue) or neither (red). **d)** Top enriched

IPA molecular functions for all genes containing WT (left) and Ames dwarf (right) hypermethylated aDMRs overlapping bivalent regions.

Figure S4: Supplementary data to Figures 2-4. a) Box-plot of mean MeDIP enrichment at shared hypermethylated aDMR bivalent regions. We observe significant increase of mean enrichment across all regions with strain and age in both instances. WTY vs. WTO (P=0.011) and DY vs. DO (P=0.011) all P<0.05 indicated by *(significance was determined using a Mann-Whitney test of median shift). **b)** Box-plot of mean MeDIP enrichment at a shared set of hypomethylated aDMR enhancer regions. We observe significant decrease in enrichment in both dwarf and WT strains with age. WTY vs. WTO and DY vs. DO all P<0.001 indicated by **(significance was determined using a Mann-Whitney test of median shift).

Figure S5: Supplementary data to Figure 6. a) Bar chart of the number of significantly differentially methylated regions (5% FDR, Fisher's Exact test, 500bp windows), between UM-HET3 young (2 months) and old (22 month) control mice (Old-Young), young and 22 month calorie restricted mice (CR-Young) and young and 22 month Rapamycin treated mice (Rapa-Young). Regions of heterogeneity (chi-squared test (<0.05) across the 4 replicates in each cohort removed. See also Supp Dataset 2. Hyper and Hypo-methylated DMRs are higher and lower in 22 month mice respectively. **b)** Scatter plot of all hypomethylated aDMRs (significant as defined in a)) between 22 month (O) and 2 month (Y) control UM-HET3 mice, showing mean percent methylation (per region) in 22 month old control mice (x-axis) and 22 month old calorie restricted mice (CR)(y-axis). Regions that are significantly different in % methylation between control and CR mice are given in red. Only regions with >=10 reads in CR were included. **c)** As b), however using percent methylation in Rapamycin treated (Rapa) instead of CR. **d)** Histogram of hypomethylated aDMR (significant as defined in a)) counts (O-Y) versus relative difference in mean % methylation per aDMR, for: (O-Y)/Y

(blue), (Rapa-Y)/Y (red) and (CR-Y)/Y (green). **e)** As b), however using all hypermethylated aDMRs between 22 month (O) and 2 month (Y) control mice. **f)** as b) however using all hypermethylated aDMRs between 22 month (O) and 2 month (Y) control mice, and % methylation in Rapamycin treated (Rapa) instead of CR. **g)** Histogram of hypermethylated aDMR (significant as defined in a)) counts (O-Y) versus relative difference in mean % methylation per aDMR, for: (O-Y)/Y (blue), (Rapa-Y)/Y (red) and (CR-Y)/Y (green). **h)** Left : Clustered feature interactions maps of spatial overlap between DMRs (significant as defined in a)) that are hypomethylated in CR-Young but not Old-Young. Red indicates an overlap between DMR and feature and blue no overlap. 1301 regions. Other 3 interaction maps as indicated. Interaction map x-axes are scaled by number of DMRs indicated.
